# Regulation of chaperone binding and nucleosome dynamics by key residues within the globular domain of histone H3

**DOI:** 10.1186/s13072-016-0066-4

**Published:** 2016-04-30

**Authors:** Sarah J. Hainer, Joseph A. Martens

**Affiliations:** Department of Molecular, Cell, and Cancer Biology, University of Massachusetts Medical School, 364 Plantation Street, LRB 560D, Worcester, MA 01604 USA; Shady Side Academy Senior School, 423 Fox Chapel Road, Pittsburgh, PA 15238 USA; Department of Biological Sciences, University of Pittsburgh, Pittsburgh, PA 15260 USA

**Keywords:** Transcription, Chromatin, *SRG1*/*SER3*, Histone chaperone, Spt6, Spt16, Spt2, Histone H3 K122, *Saccharomyces cerevisiae*

## Abstract

**Background:**

Nucleosomes have an important role in modulating access of DNA by regulatory factors. The role specific histone residues have in this process has been shown to be an important mechanism of transcription regulation. Previously, we identified eight amino acids in histones H3 and H4 that are required for nucleosome occupancy over highly transcribed regions of the genome.

**Results:**

We investigate the mechanism through which three of these previously identified histone H3 amino acids regulate nucleosome architecture. We find that histone H3 K122, Q120, and R49 are required for Spt2, Spt6, and Spt16 occupancies at genomic locations where transcription rates are high, but not over regions of low transcription rates. Furthermore, substitution at one residue, K122, located on the dyad axis of the nucleosome, results in improper reassembly and disassembly of nucleosomes, likely accounting for the transcription rate-dependent regulation by these mutant histones.

**Conclusions:**

These data show that when specific amino acids of histone proteins are substituted, Spt2, Spt6, and Spt16 occupancies are reduced and nucleosome dynamics are altered. Therefore, these data support a mechanism for histone chaperone binding where these factors interact with histone proteins to promote their activities during transcription.

**Electronic supplementary material:**

The online version of this article (doi:10.1186/s13072-016-0066-4) contains supplementary material, which is available to authorized users.

## Background

In eukaryotes, genomic DNA is packaged with an octamer of histone proteins to form nucleosomes. Nucleosomes, in turn, form a three-dimensional structure called chromatin in order to package DNA into the nucleus. This stable association of DNA with histone proteins poses a significant obstacle to many cellular processes that rely on proteins being able to interact with DNA, including transcription, DNA replication, and DNA repair (reviewed in [1–5]). Histones are small, highly conserved, positively charged proteins consisting of a folded domain that forms the nucleosome globular core and highly unstructured N- and C-terminal tails that extend out from this core. Nucleosomes are repeated along the length of DNA, with approximately 10–80 bp between each nucleosome, forming a chromatin template [6].

In general, most eukaryotic promoters are nucleosome-depleted regions (NDRs), which permit binding of transcription factors and successful transcription initiation [7–9]. Transcription initiation can be hindered when promoter DNA is wrapped into a nucleosome, which can no longer be easily recognized by DNA binding factors [10]. Transcription elongation can also be physically hindered by nucleosome occupancy in that transcription rates of RNA polymerase II (RNA pol II) are slowed due to increased pausing and backtracking [11, 12]. Therefore, the mechanisms through which eukaryotes regulate chromatin dynamics are utilized during various stages of transcription to successfully regulate gene expression.

Changes in transcription of genes are tightly correlated with changes in chromatin structure [8, 13–17]. During transcription initiation and elongation, nucleosomes are commonly evicted from promoters and coding regions. Histone chaperones, such as Asf1, Spt6, and Spt16/FACT, are factors that have been implicated in this process. These factors have been shown to interact with nucleosomes in vitro, associate with chromatin in vivo, and facilitate histone deposition, exchange, or eviction from chromatin (reviewed in [1–5, 18–21]). Removal of nucleosomes from promoters is essential for proper recruitment of RNA pol II and other initiation factors. Furthermore, removal of nucleosomes ahead of RNA pol II is essential for efficient transcription elongation while replacement of nucleosomes behind transcribing RNA pol II is essential to prevent initiation of intragenic cryptic transcripts [6, 15, 22–26]. Each histone chaperone has specificity for particular histones or portion of nucleosome and facilitates different steps in the assembly, disassembly, or exchange of histones (reviewed in [7–9, 20]).

We previously identified eight amino acids in histones H3 and H4 (H3 K122, H3 Q120, H3 V117, H3 R49, H3 S47, H3 V46, H4 R36, and H4 I46) that are required for nucleosome occupancy over highly transcribed regions of the genome, but not lowly transcribed regions [27]. Several studies have indicated that mutations at histone residues may disrupt the recruitment and/or function of histone chaperones that are directly involved in transcription-dependent nucleosome assembly [11, 12, 22, 23, 26, 28–33]. Therefore, to determine the mechanism through which these histone residues regulate nucleosome architecture, we performed chromatin immunoprecipitation (ChIP) analyses on a subset of these residue mutations (H3 K122A, Q120A, and R49A) and found that these three histone residues are required for occupancy of Spt2, Spt6, and Spt16, but not Asf1, Paf1, and Rpb3. Furthermore, we found that mutating at least one of these amino acids results in decreased rates of nucleosome disassembly and reassembly. Together, our results suggest that histones are required for the maintenance of these histone chaperones and slowed nucleosome reassembly and disassembly occurs when histones are mutated and these factors are no longer properly maintained.

## Results

**Fig. 1:**
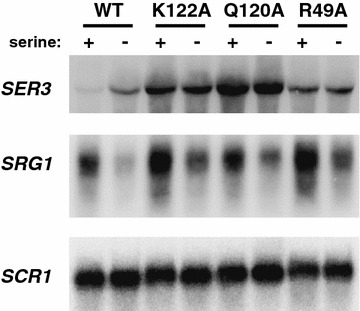
Single amino acid substitutions expressed at both *HHT1*-*HHF1* and *HHT2*-*HHF2* strongly derepress *SER3*. Northern blot analysis examining the effect of histone mutants on *SER3*, *SRG1*, and *SCR1* (loading control). Total RNA was isolated from yeast strains that were grown to a density of 1–2 × 10^7^ cells/mL in media with or without serine at 30 °C. All yeast strains were derivatives of JDY86 expressing either synthetic WT copies of histone H3 at both genomic locations (YS417) or mutants *hhts*-*K122A* (YS404)*, hhts*-*Q120A* (YS409), *hhts*-*R49A* (YS428), also expressed at both genomic locations

Serine-dependent transcription of *SRG1*, a non-coding RNA (ncRNA), represses expression of the adjacent *SER3* gene by maintaining nucleosomes over the promoter region of *SER3* [34]. We previously identified ten single histone amino acid residue substitutions in histones H3 or H4 that upregulate *SER3* with little to no effect on *SRG1* levels, in combination with one copy of the histone H3/H4 genes (*hht1*-*hhf1*) deleted [27]. Furthermore, we found histones harboring these individual point mutations result in transcription-dependent reduction of nucleosome occupancy: regions of the genome that are highly transcribed (such as *SRG1*) have decreased histone H3 occupancy, whereas regions of the genome that are lowly transcribed (such as *SER3*) show no change in histone H3 occupancy. Previously, we found that one copy of the histone H3/H4 genes is deleted, and there is a slight upregulation in *SER3* expression levels [27]. Therefore, we created a set of histone residue substitutions (H3 K122A, H3 Q120A, and H3 R49A) where both copies of the histone genes contain the same single residue substitution. In addition to reflecting more accurate levels of *SER3* expression relative to strains containing a deletion of one histone H3/H4 allele, these strains prevent any global disruption to transcription and chromatin dynamics that may occur in strains containing a histone gene deletion and permit for more straightforward interpretations of results. We concentrated on this subset of histone residue substitutions for the following reasons: (1) K122 and Q120 are found over the dyad of the nucleosome, where DNA makes a strong interaction with the histone proteins; (2) K122 is especially interesting, as we isolated three individual substitutions for this amino acid (K122A, K122R, and K122Q) during our initial screen [27], and K122 has been previously described as a binding site for the histone chaperone, Asf1 [35]; (3) H3 R49 has an alternative location at the entry/exit point of DNA wrapping around the histone octamer as well as additional phenotypes when mutated [27], compared to the other histone residues. To create strains where both histone genes contain the single histone H3 point mutation, we obtained integrating plasmids of the histone mutants (kind gift from Junbiao Dai, Tsinghua University, Beijing, China) that contain the synthetic versions of the histone mutants, targeted to the *HHT1*-*HHF1* locus. After integrating each mutation into the second histone locus, we performed Northern blot analysis to examine the effect of the newly created strains on *SER3* and *SRG1* expression levels (Fig. 1). Each residue substitution resulted in increased *SER3* mRNA levels in the presence or absence of serine.

### Histone mutants reduce Spt2, Spt6, and Spt16 occupancy at *SRG1/SER3*

**Fig. 2:**
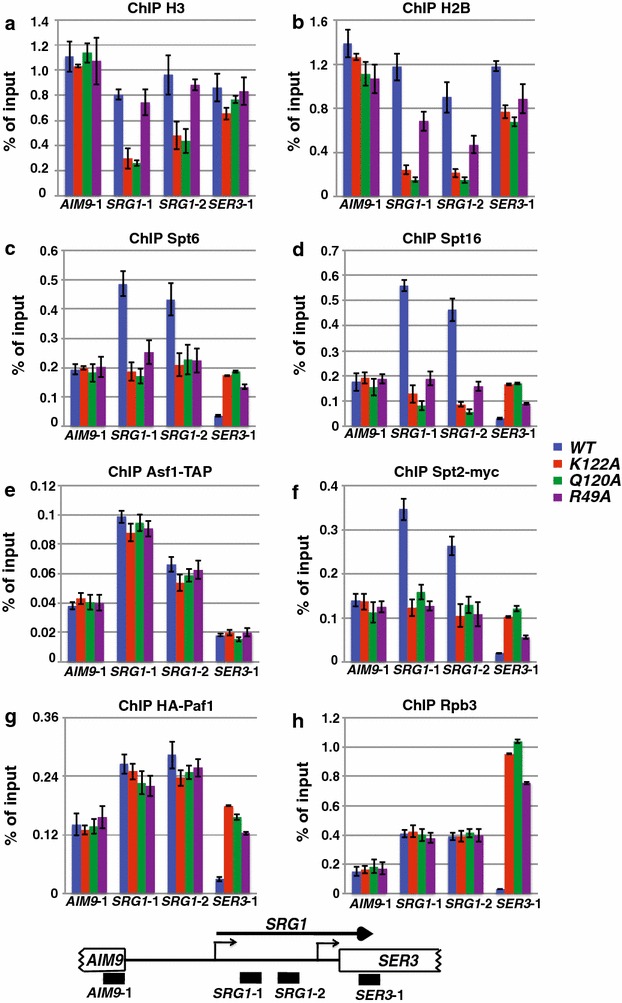
Spt2, Spt6, and Spt16 have reduced occupancy over *SRG1*, a highly transcribed RNA, in histone mutations. ChIP of histone H3 (**a**), H2B (**b**), Spt6 (**c**), Spt16 (**d**), Spt2-13myc (**f**), HA-Paf1 (**g**), or Rpb3 (**h**) was performed on chromatin isolated from strains expressing *HHTS*-*HHFS* alleles (YS454-YS456) or the indicated histone mutant alleles (YS458-YS462, YS465, YS471, YS472, and YS474) that were grown in YPD at 30 °C. *HHTS*-*HHFS* alleles are a synthetic histone gene sequence previously developed [52] and replacing each the *HHT1*-*HHF1* and *HHT2*-*HHF2* alleles. ChIP of Asf1-TAP (**e**) was performed on chromatin prepared from strains expressing *HHTS*-*HHFS* alleles (YS493-YS495) or the indicated histone mutant alleles (YS504-YS506, YS518, YS519, YS521, YS525-YS527) that were grown in YPD at 30 °C. The amount of immunoprecipitated DNA was determined by qPCR and is shown as a percentage of the input material and represents the mean ± SEM of three biological replicate experiments. Below the* graphs* is a schematic of the *SRG1/SER3* locus with* black bars* corresponding to the regions amplified by qPCR

Previously, we found that substitution of H3 K122, Q120, or R49 to alanine results in reduced nucleosome occupancy over highly, but not lowly, transcribed regions of the genome [27]. Furthermore, we found that two histone chaperones, Spt6 and Spt16, play important roles in the regulation of *SRG1/SER3* transcription: temperature-sensitive alleles of either factor result in misregulation of *SER3* [34]. Therefore, we considered the possibility that these mutant histones fail to recruit histone chaperones normally to transcribed regions, which may account for the defects observed in transcription-coupled nucleosome occupancy. To test this possibility, we performed chromatin immunoprecipitation (ChIP) experiments to assess the occupancy of histones (H3 and H2B) and binding of histone chaperones (Spt6, Spt16, and Asf1) across the *SRG1/SER3* transcription unit (Fig. 2a–e).

In general, we detected reduced occupancy of Spt6 and Spt16 that paralleled the loss of histone H3 and H2B occupancy across *SRG1* in these histone mutant strains (compare Fig. 2a, b with c, d). Interestingly, one of the histone mutants, H3 R49A, showed a larger decrease in histone H2B occupancy compared to histone H3 occupancy. This may indicate maintenance of H3/H4 tetramers, or even a hexasome where only one H2A/H2B dimer has been lost [36, 37]. Asf1 is another histone chaperone and interacts with histones via an interface that includes H3 K122 [35]. Previously, our laboratory determined that Asf1 plays only a minor role in regulating *SER3* expression and we found that deleting Asf1 does not alter nucleosome occupancy over the *SER3* promoter (data not shown). Therefore, to test whether point mutations at these three histone residues results in specific depletion of chaperones required for gene regulation (such as Spt6 and Spt16), or results in depletion of all interacting factors, we performed ChIP-qPCR of Asf1-TAP over *SRG1/SER3* (Fig. 2e). We found that Asf1 occupancy is not significantly altered over this region, supporting our data that Asf1 is not responsible for regulating *SER3.* Furthermore, these data indicate the single substitutions at these three histone residues result in loss of specific factors required for regulating gene expression, such as Spt6 and Spt16.

To investigate this mechanism further, we assayed the roles of these amino acids in regulating the binding of factors that may act upstream of Spt6 and Spt16 to promote recruitment of these histone chaperones to chromatin: Spt2 and Paf1 [38, 39]. Spt2 is an HMG-like protein that can interact with DNA [40], so it is possible that this protein may interact with nucleosomal DNA, which is then loosened to provide a surface for histone chaperones to interact. Therefore, we tested whether these three histone mutants also alter occupancy of Spt2 at *SRG1/SER3*. We performed ChIP-qPCR of Spt2-Myc over this locus (Fig. 2f) and consistent with our hypothesis found that the three histone mutants result in decreased Spt2 occupancy specifically over *SRG1*. Previously, we found that the Paf1 transcription elongation complex is required for regulation of *SER3* expression through maintaining nucleosome occupancy over the *SER3* promoter [38]. Therefore, we tested whether the histone mutants alter Paf1 occupancy over the *SER3* promoter through ChIP of HA-Paf1 (Fig. 2g). We found that the histone mutants result in only a slight decrease in Paf1 occupancy, but this minor decrease likely cannot account for the more dramatic decrease observed in Spt6 and Spt16 occupancy in these histone mutants.

Because many of these factors strongly colocalize with RNA pol II across transcribed genes, we tested whether decreased occupancy of these factors might be indirect due to a decrease in RNA pol II occupancy at *SER3*. To this end, we performed ChIP analysis of Rpb3, a subunit of RNA pol II, over *SRG1/SER3* (Fig. 2h). Consistent with our Northern analysis (Fig. 1), we found that these histone mutants do not cause a decrease in RNA pol II occupancy relative to cells expressing wild-type (WT) histones.

As a control, we tested the effect of these mutants on histone H3, H2B, Spt2, Spt6, Spt16, Paf1, and Asf1 global protein levels by Western analysis (Additional file 1: Fig. S1). All three histone mutants express levels of these proteins indistinguishable from WT strains. Taken together, these data indicate that the amino acids defined by these mutations are required to specifically maintain occupancy of the Spt2, Spt6, and Spt16, but not Asf1 or Paf1, across *SRG1*, a highly transcribed RNA.

### Histone mutants reduce Spt2, Spt6, and Spt16 occupancy at highly transcribed genes

**Fig. 3:**
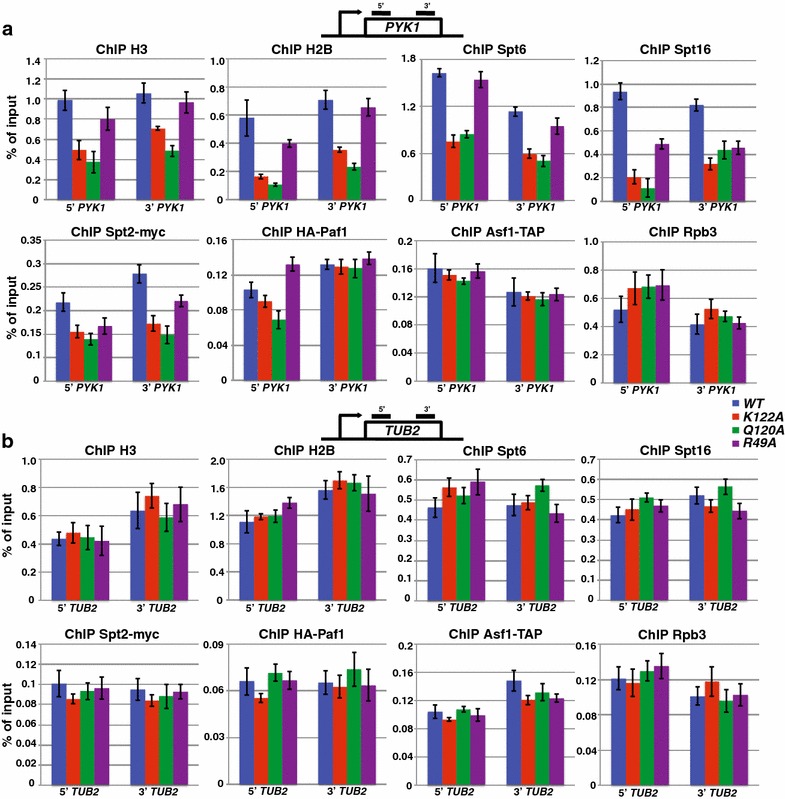
Histone mutations result in decreased Spt2, Spt6, and Spt16, occupancy over highly transcribed, but not lowly transcribed, genes. **a** ChIP analysis was performed on chromatin prepared from strains expressing *HHTS*-*HHFS* alleles (YS454-YS456, YS493-YS495) or the indicated histone mutant alleles (YS458-YS462, YS465, YS471, YS472, YS474, YS504-YS506, YS518, YS519, YS521, YS525-YS527) that were grown in YPD at 30 °C. The amount of immunoprecipitated DNA was determined by qPCR and is shown as a percentage of the input material and represents the mean ± SEM of three biological replicate experiments. Factor occupancy was measured within the coding region of a highly transcribed gene, *PYK1*. The regions assayed by qPCR are marked with the* black bars* in the diagram provided for the gene. **b** Factor occupancy at *TUB2,* a lowly transcribed gene, was determined as described in **a**

To investigate whether the histone mutants that reduce Spt2, Spt6, and Spt16 occupancies across *SRG1* have a general defect in transcription-coupled occupancy of these factors, we measured the occupancy of H3, H2B, Spt6, Spt16, Spt2, Paf1, Asf1, and Rpb3 across the coding sequences of a subset of yeast genes by ChIP (Fig. 3, Additional file 2: Fig. S2, Additional file 3: Fig. S3). At three highly transcribed genes, *PMA1* (100 mRNA/hr), *PYK1* (95 mRNA/hr), and *ADH1* (125 mRNA/hr) [41], histone H3, histone H2B, Spt2, Spt6, and Spt16 levels were reduced in all three of the histone mutants, similar to our observations over *SRG1* (Fig. 3a, Additional file 2: Fig. S2). Conversely, the occupancies of histones H3 and H2B, Spt2, Spt6, and Spt16 at three lowly transcribed genes *GAL1* (repressed), *TUB2* (12 mRNA/hr), and *CYC1* (10 mRNA/hr) [41], were unaffected in the mutants (Fig. 3b, Additional file 3: Fig. S3), similar to what was observed over *AIM9* and *SER3*, two other lowly transcribed genes (Fig. 2). The changes in Spt2, Spt6, and Spt16 binding occur in the absence of any change to RNA pol II binding (Rpb3 ChIP) to these regions (Fig. 3, Additional file 2: Fig. S2, Additional file 3: Fig. S3). Similar to our analysis at *SRG1/SER3*, we examined the effect of the histone mutants on both Paf1 and Asf1 occupancy and found that the occupancy of neither of these factors was significantly altered relative to WT (Fig. 3, Additional file 2: Fig. S2, Additional file 3: Fig. S3). Taken together, these data demonstrate that these three histone residues are generally required to maintain both nucleosome occupancy and specific histone chaperone occupancy at highly transcribed genes.

### Histone mutants reduce in vivo interactions with Spt2, Spt6, and Spt16

We next examined whether the reduction seen by ChIP of Spt2, Spt6, and Spt16 in these three histone mutants is due to decreased interaction of Spt2, Spt6 and/or Spt16 with the mutant histones. To this end, we TAP-tagged either Spt2, Spt6, or Spt16 in strains expressing either WT histone alleles or one of the three histone mutations and performed a TAP pull-down assay to test whether the histone amino acid substitutions reduced interactions with these factors (Additional file 4: Fig. S4). We observed that Spt2, Spt6, and Spt16 each exhibited reduced interaction with each of the three histone mutants relative to WT. These data support the hypothesis that substitution of histone H3 K122, H3 Q120, or H3 R49 results in decreased interaction of histones with Spt2, Spt6, and Spt16 on chromatin.

### Genetic relationship between histone residue substitutions and *SPT2, SPT6*, and *SPT16* mutations

**Fig. 4:**
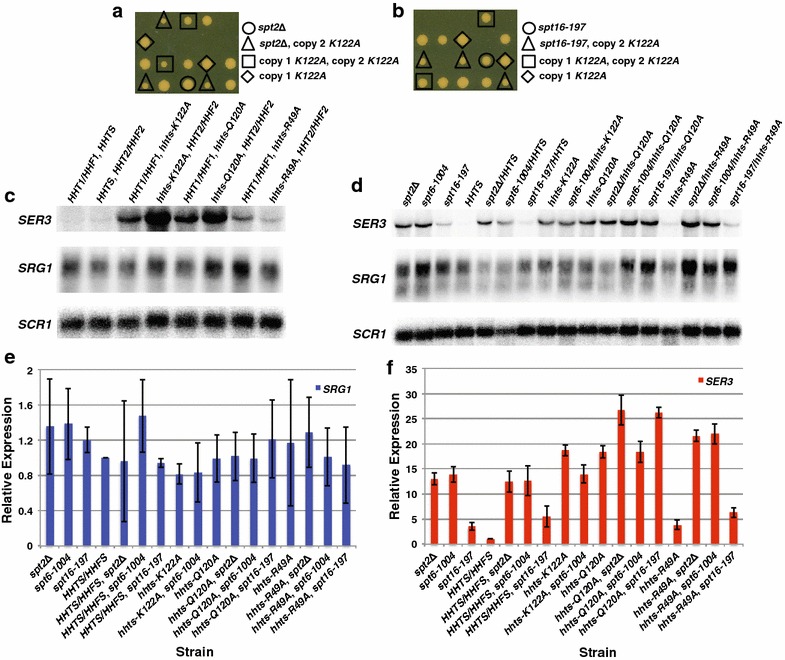
Genetic relationship between histone substitutions and either *spt2*Δ, *spt6*-*1004*, or *spt16*-*197.* Tetrad analysis of *hhts*-*K122A/hhts*-*K122A* with *spt2*Δ (YS641 X YS642; **a**) or *spt16*-*197* (YS640 X YJ780; **b**). Tetrads are vertical, where those* circled* indicate either *spt2*Δ or *spt16*-*197* alleles alone, those surrounded by a* triangle* express either *spt2*Δ or *spt16*-*197* in combination with *hhts*-*K122A* expressed at the *HHT2/HHF2* locus, those surrounded by a* square* express *hhts*-*K122A* at both histone loci, those surrounded by a* diamond* express *hhts*-*K122A* at the *HHT1/HHF1* locus, those which have a spore growing and are not indicated are WT for *SPT2* or *SPT16* and the histone loci, and those spores which do not grow are expressing either *spt2*Δ or *spt16*-*197* and either *hhts*-*K122A* at both histone loci, or only at the *HHT1/HHF1* locus. **c** Northern blot analysis examining the effect of single allele histone mutants on *SER3*, *SRG1*, and *SCR1* (loading control). Total RNA was isolated from YS542, YS545, YS548, YS551, YS554, YS557, YS560, and YS563 that were grown to a density of 1–2 × 10^7^ cells/mL in YPD at 30 °C. **d** Northern blot analysis examining the effect of histone mutants in combination with *spt2*Δ*, spt6*-*1004* or *spt16*-*197* on *SER3*, *SRG1*, and *SCR1* (loading control). Total RNA was isolated from FY346, FY2180, YJ804, YS404, YS409, YS417, YS428, YS591, YS594, YS597, YS600, YS601, YS604, YS606, YS609, YS612, and YS614 that were grown to a density of 1–2 × 10^7^ cells/mL in YPD at 30 °C. **e**, **f** Quantitation of Northern analyses. *SRG1* (**e**) and *SER3* (**f**) RNA levels for the histone mutants are normalized to the *SCR1* loading control and are relative to the *SRG1* and *SER3* RNA levels measured in control *HHTS*-*HHFS* strains (arbitrarily set to 1). Each* bar* represents the mean ± SEM from three independent experiments strains generated by genetic crosses (YS591-YS619), except the *spt16*-*197/hhts*-*Q120A* strains, of which two independent strains were used, and the experiment was performed in triplicate (YS604, YS605)

To further understand the relationship between the histone amino acids and Spt2, Spt6, and Spt16, we performed yeast crosses and dissected tetrads to generate haploid strains expressing WT, K122A, Q120A, or R49A histones with either *spt2*Δ, *spt6*-*1004*, or *spt16*-*197* alleles. Interestingly, while we were able to generate the majority of these strains, *K122A* in combination with *spt2*Δ or *spt16*-*197* was synthetically lethal (Fig. 4a, b). Furthermore, while we obtained tetrads where copy 1 of histone H3 was WT and copy 2 was *K122A* with either *spt2*Δ or *spt16*-*197* alleles, we were unable to generate cells in which copy 1 was *K122A* and copy 2 was WT with the *spt2*Δ or *spt16*-*197* alleles, suggesting that this configuration is also synthetically lethal (Fig. 4a, b). To address the differing contribution of the two histone H3/H4 loci, we performed Northern blot analysis in strains expressing one WT allele and one allele containing the histone substitution (Fig. 4c). We found that when copy 1 of the histone alleles carried either *K122A* or *Q120A*, the effect on *SER3* expression was much stronger, which suggests our inability to generate strains harboring copy 1 *K122A* with *spt2*Δ or *spt16*-*197* is due to a differential effect from the two histone loci.

With the double mutant strains we were able to generate, we performed Northern blot analysis to determine whether the histone mutants in combination with the *spt2*, *spt6*, or *spt16* mutants had any epistatic relationship (Fig. 4d–f). Importantly, the WT synthetic histone strain did not alter the effects *spt2*Δ, *spt6*-*1004*, or *spt16*-*197* have on *SRG1*/*SER3* RNA levels seen previously (Fig. 4d–f, [34]). Interestingly, while *K122A* or *Q120A* did not have any combinatorial effects on *SER3* expression with *spt6*-*1004*, Q120A in combination with either *spt2*Δ or *spt16*-*197* did have an additive effect on *SER3* expression. Conversely, *R49A* in combination with all *spt2*Δ or *spt6*-*1004* resulted in an additive effect on *SER3* expression, but in combination with *spt16*-*197* did not have any combinatorial effects on *SER3* expression. Together, these data reveal a genetic relationship between histone residues and Spt2, Spt6, and Spt16. While there is an interaction between histone chaperones and histone H3 involving these amino acids, these genetic data suggest that the histone mutations may have some additional effect on transcription that is independent of the histone chaperones.

### H3 K122A results in decreased nucleosome reassembly and disassembly

**Fig. 5:**
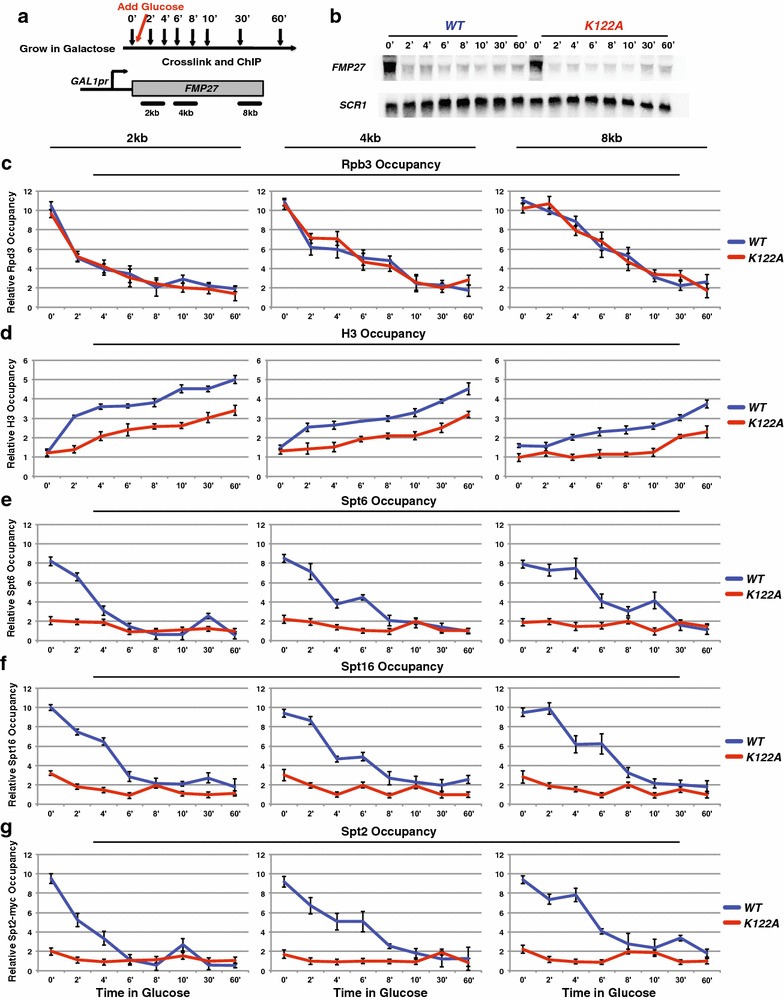
H3 K122A results in a reduced rate of histone reassembly over *FMP27*. **a** Diagram depicting the experimental procedure upon repression of *GAL1*pr-*FMP27*. **b** Northern blot analysis examining the effect of WT (YS475) and K122A (YS585) on *FMP27* expression during transcription repression where *SCR1* serves as a loading control. Rpb3 (**c**), H3 (**d**), Spt6 (**e**), Spt16 (**f**), and Spt2 (**g**) ChIP was performed on chromatin isolated from strains containing *HHTS*-*HHFS* alleles (YS475, YS477, and YS478) or *hhts*-*K122A* mutant alleles (YS585-YS587), expressing *GAL1*pr-*FMP27* that were grown in YPGal at 30 °C to approximately 1 × 10^7^ cells/mL (0′), then repressed by adding glucose and time points were taken, as shown (**a**). The amount of immunoprecipitated DNA was determined by qPCR as a percentage of the input material normalized to a control region in chromosome V (which is unchanged between WT and K122A) and represents the mean ± SEM of three biological replicate experiments

**Fig. 6:**
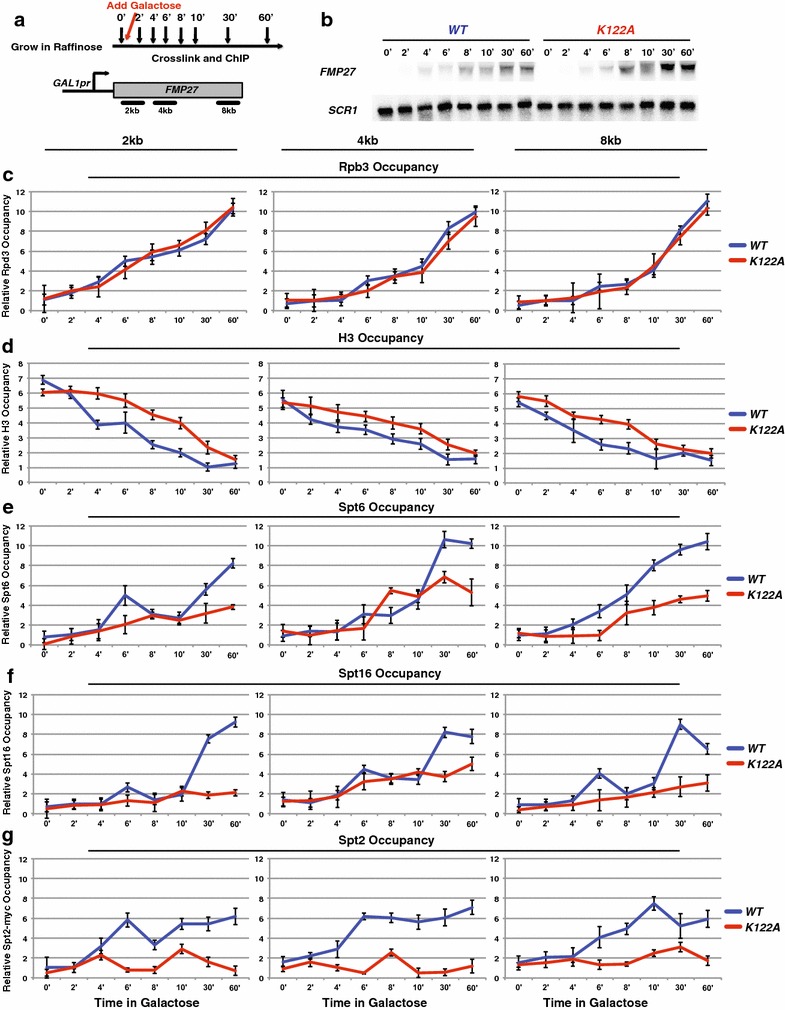
H3 K122A results in a reduced rate of histone disassembly over *FMP27*. **a** Diagram depicting the experimental procedure upon induction of *GAL1*pr-*FMP27*. **b** Northern blot analysis examining the effect of WT (YS475) and K122A (YS585) on *FMP27* expression during transcription induction where *SCR1* serves as a loading control. Rpb3 (**c**), H3 (**d**), Spt6 (**e**), Spt16 (**f**), and Spt2 (**g**) ChIP was performed on chromatin isolated from strains containing *HHTS*-*HHFS* alleles (YS475, YS477, and YS478) or *hhts*-*K122A* mutant alleles (YS585-YS587) expressing *GAL1*pr-*FMP27* that were grown in YPRaff at 30 °C to approximately 1 × 10^7^ cells/mL (0′), then induced by adding galactose and time points were taken, as shown (**a**). The amount of immunoprecipitated DNA was determined by qPCR as a percentage of the input material normalized to a control region in chromosome V (which is unchanged between WT and K122A) and represents the mean ± SEM of three biological replicate experiments

A possible scenario for how the histone residues may be functioning to regulate transcription-coupled nucleosome dynamics is that a reduction in DNA affinity through histone residue mutations may slow nucleosome reassembly after passage of RNA pol II. This could account for our contrasting observations between lowly and highly transcribed regions of the genome. This hypothesis is supported by in vitro studies examining how RNA pol II overcomes a nucleosome barrier [42–44]. At lowly transcribed genes, a nucleosome should have sufficient time to reassemble prior to the passage of the next RNA pol II so the density of nucleosomes will not be affected. However, at highly transcribed genes, nucleosomes may only be partially assembled before being disassembled during passage of the next RNA pol II molecule, resulting in reduced nucleosome occupancy at these genes [42–44]. To test this hypothesis, we adopted a strategy that has been previously described [45], where a long gene, *FMP27* (also named *YLR454W*), is under the control of the inducible *GAL1* promoter (*GAL1*pr). Using this construct, one can either turn off the promoter to follow the last wave of RNA pol II, and subsequently nucleosome reassembly (Fig. 5), or turn on the promoter to follow the recruitment of RNA pol II and subsequently nucleosome disassembly (Fig. 6). Using this strategy, we examined the effect of *K122A* on nucleosome reassembly and disassembly compared to control WT cells. During both induction and repression of *FMP27,* the expression of the construct followed the predicted profile, given the carbon source provided (Fig. 5b: gene turns off following glucose repression and Fig. 6b: gene turns on following galactose induction).

When we examined the reassembly of nucleosomes by turning transcription off at this gene, the *K122A* mutant resulted in slowed reassembly of the nucleosomes compared to WT, even though RNA pol II kinetics at this region were similar (Fig. 5c, d). Similar to the previous findings [45], the occupancies of Spt6 and Spt16 mirrored those of RNA pol II in the WT strain throughout the time course (Fig. 5e, f). Under these conditions, low levels of Spt6, Spt16, and Spt2 were observed in the *K122A* mutant (Fig. 5e–g), which is consistent with our previous findings of low occupancy for these factors over highly transcribed genes.

We next examined the disassembly of nucleosomes by inducing transcription and found the RNA pol II kinetics was not significantly affected in the *K122A* mutant strain compared to WT (Fig. 6c). When we examined the occupancy of histones over this region upon induction, there was a slight delay in the reduction of histone occupancy in the *K122A* strain compared to WT (Fig. 6d). We next examined histone chaperone recruitment and found that in the *K122A* mutant strain, Spt6, Spt16, and Spt2 were recruited less rapidly and to lower amounts compared to occupancies observed in WT strains (Fig. 6e–g). Taken together, these data suggest that, in addition to RNA pol II, H3 K122 is required for maximal Spt6, Spt16, and Spt2 occupancy at transcribed genes. When these factors are not properly recruited, nucleosome reassembly is impaired.

## Discussion

Previously, we identified specific histone amino acids that are required for transcription-dependent nucleosome occupancy [27]. We hypothesized that disassembly and/or reassembly might be slowed in the histone mutants due to reduced occupancy and/or function of histone chaperones, such as Spt6 or Spt16. To test this, we examined whether the histone mutants were altering histone chaperone occupancy and function. We found that both Spt6 and Spt16, but not Asf1, have reduced occupancy, specifically over highly transcribed regions, in three of the previously identified histone mutants (H3 K122A, H3 Q120A, and H3 R49A). Interestingly, Spt2 occupancy is also decreased in these histone mutants, supporting the possibility that these mutations prevent the unwrapping of nucleosomal DNA by Spt2, thereby denying access of nucleosomal histones to the affected chaperones. Conversely, Spt2 has been shown to have reduced recruitment when Spt6 is mutated, and therefore, the loss of Spt2 could be indirect [40]. Furthermore, the loss of all three of these factors could be either direct, due to the histone point mutations, or indirect, due to the resulting loss of histone occupancy. Regardless of whether the effect is direct or indirect, the histone mutations result in reduced histone chaperone occupancy.

To mechanistically address how the defect in histone chaperone occupancy is affecting nucleosome dynamics at highly, and not lowly, transcribed regions of the genome, we utilized an established system in which the expression of a long gene is inducible based on the sugar source available in the cell [45]. Our data demonstrate that, while in a WT strain similar occupancy patterns to RNA pol II are observed for both Spt6 and Spt16, RNA pol II alone is not sufficient for the maintenance and/or recruitment of these histone chaperones. Due to the near-complete lack of occupancy of these histone chaperones in the *K122A* strain, we hypothesize that K122 (and likely Q120 and R49) is required for the maintenance of Spt6 and Spt16 occupancy over transcribed regions. These data argue against the traditional view that RNA pol II is simply recruiting these factors and rather supports the important interactions made between histones and chromatin regulatory factors during transcription. Rather, these results suggest a requirement for both transcription and interaction with histones to effectively recruit and maintain the histone chaperones. It is possible that the histone chaperones are recruited to DNA by RNA pol II during transcription elongation and then retention of these proteins depends on interactions with histones and further experiments are necessary to distinguish recruitment from maintenance. Based on the nucleosome reassembly defect observed in *K122A*, and the lack of histone chaperone occupancy, our data support the hypothesis that *K122A* causes slowed reassembly of nucleosomes due to the loss of histone chaperone binding. However, an alternative explanation that our data do not discount could be that the decrease in nucleosome occupancy causes a decrease in histone chaperone occupancy. These two models represent a conundrum of which came first: the decreased nucleosome or decreased chaperone, and our data cannot distinguish between these two possible mechanisms. While we hypothesize that *Q120A* would result in similar defects in nucleosome reassembly and disassembly, H3 R49A increases the rate of nucleosome repositioning by RSC in vitro [46], and therefore, this mutation may differ in its regulatory mechanism for histone chaperone occupancy.

Previous studies have demonstrated that K122 is acetylated through p300/CBP in mammalian cells, and this modification plays an important role in stimulating transcription [47, 48]. Furthermore, recent studies have shown that Rtt101 ubiquitylates K122 in a H3 K56 acetyl-dependent manner, and this ubiquitylation is important for nucleosome assembly in yeast [49]. Based on these studies, it would be interesting to determine the extent to which acetylation and/or ubiquitylation is necessary for the nucleosome reassembly defect we observe in yeast.

While the actual affinity of the mutant nucleosomes to Spt2, Spt6, and Spt16 remains untested, we show that interaction of Spt2, Spt6, and Spt16 with histones is reduced by amino acid substitutions in K122, Q120, and R49 of histone H3. Taken together, these studies support a role for these histone residues in properly maintaining occupancy of Spt2, Spt6, and Spt16 in order for these factors to promote proper assembly of transcribing nucleosomes. Overall, these data support a novel mechanism for how histone chaperones are recruited to and maintained on chromatin during transcription.

## Conclusions

We report a mechanistic role for specific histone H3 residues in directly regulating transcription-coupled nucleosome assembly by histone chaperones. Utilizing previously identified histone amino acids that are critical for maintenance of chromatin architecture, we found that these three residues are required for optimal chromatin binding by the histone chaperones Spt6 and Spt16/FACT and the HMG-like protein Spt2, specifically at highly transcribed genes. Furthermore, H3 K122A results in impaired nucleosome reassembly and disassembly, which may be either due to or causing the loss of histone chaperone occupancy. These data provide insight into the histone-based regulation of histone chaperone binding and function.

## Methods

### Strains and media

All *S. cerevisiae* strains used in this study (Additional file 5: Table S1) are isogenic with a *GAL2*+ derivative of S288C [50]. Strains were constructed using standard genetic crosses or by transformation. Tagged versions of Spt2 and Paf1 have been previously described [40, 51]. Asf1-TAP, Spt2-TAP, Spt6-TAP, and Spt16-TAP strains were validated from the TAP-tag collection (Open Biosystems) and amplified from this strain to tag Asf1, Spt2, Spt6, and Spt16, respectively, in the S288C strain background. Synthetic histone strains were created by one-step integration of plasmids expressing synthetic histone genes targeted for *HHT1/HHF1* tagged with a hygromycin-resistant cassette (kind gift from J. Dai, Tsinghua University) into JDY86 strains expressing the same synthetic histone gene sequence at *HHT2/HHF2* [52]. Briefly, plasmids were linearized with BciVI and transformed into the JDY86 strain harboring the specific point mutation. Transformants were selected on YPD media containing 200 μg/mL of hygromycin and confirmed through PCR and sequencing. Strains were grown in the following media as indicated in the figure legends: YPD (1 % yeast extract, 2 % peptone, 2 % glucose), YPGal (1 % yeast extract, 2 % peptone, 2 % galactose), YPRaff (1 % yeast extract, 2 % peptone, 2 % raffinose), and synthetic complete medium with 1 mM serine (+serine) or without serine (−serine).

### Northern analysis

Cells were grown to 1–2 × 10^7^ cells/mL and separated on a 1 % formaldehyde-agarose gel. Total RNA isolation and Northern analysis were performed as previously described [53]. RNA was transferred to a Gene Screen membrane (Perkin-Elmer) and hybridized with radiolabeled probes generated by random-primed labeling of PCR fragments for *SRG1* (−424 to −123 relative to *SER3* ATG)*, SER3* (+1378 to +1606), *FMP27* (+1982 to +2296), and *SCR1* (−182 to +284) that were amplified from genomic DNA. RNA levels were quantified using a PhosphorImager (Instant Imager, Packard Co.) and normalized to the *SCR1* loading control.

### Western analysis

Whole cell extracts (WCE) were prepared from cells grown in YPD at 30 °C to approximately 3 × 10^7^ cells/mL using trichloroacetic acid as previously described [54]. Equal amounts of WCE were separated by 12.5 or 15 % acrylamide SDS-PAGE, transferred to nitrocellulose (Whatman), and assayed by immunoblotting. The antibodies used to detect H3, H2B, Spt6, Spt16, Pob3, TAG, HA, Myc, and G6DPH were as follows: anti-H3 (1:30,000, described in [55] 1), anti-H2B (1:2500, Active Motif), anti-Spt6 (1:1000, gift from Tim Formosa), anti-Spt16 (1:500, gift from Tim Formosa), anti-Pob3 (1:2000, gift from Tim Formosa), anti-TAP (1:2000, Sigma), anti-HA (1:2000, Santa Cruz), anti-Myc (1:1000, Santa Cruz), and anti-GAPDH (1:50,000, Sigma). After incubation with HRP-conjugated IgG or secondary antibody (1:5000; GE Healthcare), the immunoreactive proteins were visualized by enhanced chemiluminescence detection (Perkin-Elmer) using a Kodak image station 440CF. Protein levels were calculated by measuring their signal intensities in these Western blots using Kodak ID 3.6 software and normalizing these values to those obtained for the G6PDH control.

### Chromatin immunoprecipitation (ChIP)

For ChIP over galactose-induced *GAL1*pr-*FMP27*, cells were grown in YPRaff to approximately 1 × 10^7^ cells/mL and then 2 % galactose was added at time zero. For ChIP over galactose-repressed *GAL1*pr-*FMP27*, cells were grown in YPGal to approximately 1 × 10^7^ cells/mL and then 2 % glucose was added at time zero. For all other ChIP experiments, cells were grown in YPD at 30 °C to 1–2 × 10^7^ cells/mL. Chromatin was prepared as previously described [56]. Histone H3, histone H2B, Spt6, Spt16, Rpb3, Spt2-Myc, or HA-Paf1 were immunoprecipitated by incubating sonicated chromatin overnight at 4 °C with 1 μL anti-histone H3 (described previously [55]), 3 μL anti-histone H2B (Active Motif), 1 μL anti-Spt6 (gift from Tim Formosa), 1 μL anti-Spt16 (gift from Tim Formosa), 2.5 μL anti-Rpb3 (Neoclone), 1 μL anti-Myc (Santa Cruz), 1 μL anti-HA (Santa Cruz), antibodies and then adding IgG-sepharose beads (GE Healthcare) for 2 h at 4 °C. Asf1-TAP was immunoprecipitated by incubating sonicated chromatin for 4 h at 4 °C with IgG-sepharose beads (GE Healthcare). Dilutions of input DNA and immunoprecipitated DNA were analyzed by qPCR reactions. Primer sets that amplify the following regions were used to measure occupancy by qPCR: *PYK1* (5′: +62 to +164, 3′: +1173 to +1279)*, PMA1* (5′: +691 to +794, 3′: +1689 to +1791)*, ADH1* (+845 to +943)*, CYC1* (+122 to +217)*, TUB2* (5′: +105 to +202, 3′: +1083 to +1189), *GAL1* (5′: +79 to +175, 3′: +1366 to +1487), *FMP27* (pr: −194 to +35, 2 kb: +1986 to +2199, 4 kb: +4069 to +4268, 6 kb: +5901 to +6074, 8 kb: +7701 to +7850). ChIP signals for each gene were normalized to a No ORF control template, which is located within a region of chromosome V that lacks open reading frames [57].

### Quantitative PCR (qPCR)

All qPCR data for ChIP assays were obtained using a StepOnePlus Real-time PCR system, SYBR green reagent (Fermentas), and the indicated primers. Calculations were performed using Pfaffl methodology [58].

### TAP-tag pull-down assay

To examine the interaction between Spt2, Spt6, or Spt16 with histone H3 or histone mutant strains expressing either WT synthetic histones or one of the mutations with TAP-tagged versions of either Spt2, Spt6, or Spt16 (YS482, YS485, YS490, YS497, YS501, YS508, YS511, YS514, YS522, YS538, YS565, YS570) were grown in YPD medium to approximately 3–4 × 10^7^ cells/mL. Whole cell extracts were made by glass bead lysis in lysis buffer (20 mM HEPES, pH 7.4, 100 mM sodium acetate, 2 mM magnesium acetate, 100 mM sodium acetate, 10 mM EDTA, 10 % glycerol, 1 mM dithiothreitol, and PMSF). Extracts were clarified by centrifugation, and 5 mg of protein was then incubated at 4 °C for 3.5 h with 30 μL IgG conjugated to sepharose beads (GE Healthcare). Bound complexes were washed twice with lysis buffer containing 400 mM sodium chloride. Precipitates were resolved on a 12.5 % SDS-PAGE and analyzed by immunoblotting with antibodies specific to histone H3 (1:30,000 dilution; [55]) or TAP (1:2000; Sigma).

## Additional files

10.1186/s13072-016-0066-4 Histone residue substitutions do not alter total protein levels. (**a**) Western analysis examining the effect of histone mutants on total histone H3, H2B, Spt6, Spt16, Pob3, Asf1-TAP, HA-Paf1, and Spt2-Myc protein levels. Strains expressing the indicated histone alleles (YS417, YS404, YS409, YS428, YS454, YS458, YS462, YS471, YS493, YS504, YS518, YS525) were grown to approximately 3 × 10^7^ cells/mL in YPD at 30 °C. Proteins were extracted with trichloroacetic acid and subjected to Western analysis using anti-H3, anti-H2B, anti-Spt6, anti-Spt16, anti-Pob3, anti-PAP, anti-HA, anti-Myc, and anti-G6PDH (loading control). (**b**) Quantitation of Western analysis, where similar results were obtained for three independent experiments and WT was arbitrarily set to 1 and error bars represent the mean ± SEM of three biological replicate experiments.
10.1186/s13072-016-0066-4 Histone mutations result in decreased Spt2, Spt6, and Spt16, occupancy over highly transcribed genes. (**a**) ChIP analysis was performed on chromatin prepared from strains expressing *HHTS*-*HHFS* alleles (YS454-YS456, YS493-YS495) or the indicated histone mutant alleles (YS458-YS462, YS465, YS471, YS472, YS474, YS504-YS506, YS518, YS519, YS521, YS525-YS527) that were grown in YPD at 30 °C. The amount of immunoprecipitated DNA was determined by qPCR and is shown as a percentage of the input material and represents the mean ± SEM of three biological replicate experiments. Factor occupancy was measured within the coding region of a highly transcribed gene, *PMA1*. The regions assayed by qPCR are marked with the black bars in the diagram provided for the gene. (**b**) Factor occupancy at *ADH1,* a highly transcribed gene, was determined as described in **a**.
10.1186/s13072-016-0066-4 Histone mutations do not alter Spt2, Spt6, and Spt16, occupancy over lowly transcribed genes. (**a**) ChIP analysis was performed on chromatin prepared from strains expressing *HHTS*-*HHFS* alleles (YS454-YS456, YS493-YS495) or the indicated histone mutant alleles (YS458-YS462, YS465, YS471, YS472, YS474, YS504-YS506, YS518, YS519, YS521, YS525-YS527) that were grown in YPD at 30 °C. The amount of immunoprecipitated DNA was determined by qPCR and is shown as a percentage of the input material and represents the mean ± SEM of three biological replicate experiments. Factor occupancy was measured within the coding region of a *GAL1*. (**b**) Factor occupancy at *CYC1,* a lowly transcribed gene, was determined as described in **a**.
10.1186/s13072-016-0066-4 Histone mutants cause decreased interaction with Spt2, Spt6, and Spt16. Pull down of Spt2-TAP (**a**), Spt6-TAP (**b**), or Spt16-TAP (**c**) in strains expressing WT, H3 K122A, H3 Q120A, or H3 R49A histone alleles. Extracts from strains expressing WT (YS482, YS485, YS490), K122A (YS497, YS538, YS501), Q120A (YS508, YS511, YS514) or R49A (YS565, YS570, YS522) were incubated with IgG sepharose. Immunoblot analysis was performed to assess the presence of histone H3 and TAP-Spt2, Spt6, or Spt16 in the pull-down fractions (lanes 5–8). Lanes 1–4 in each blot represent 1 % of input material.
10.1186/s13072-016-0066-4 *Saccharomyces cerevisiae* strains used in this study.

## Abbreviations

ChIP: chromatin immunoprecipitation
TSS: transcription start site
NDR: nucleosome-depleted region
RNA pol II: RNA polymerase II
ncRNA: non-coding RNA
ncDNA: non-coding DNA
WT: wild-type
