# Supplementary figures and images for: Regulation of chaperone binding and nucleosome dynamics by key residues within the globular domain of histone H3
Source: Epigenetics Chromatin. 2016 Apr 30;9:17. doi: 10.1186/s13072-016-0066-4 (PMC4851828; doi:10.1186/s13072-016-0066-4)

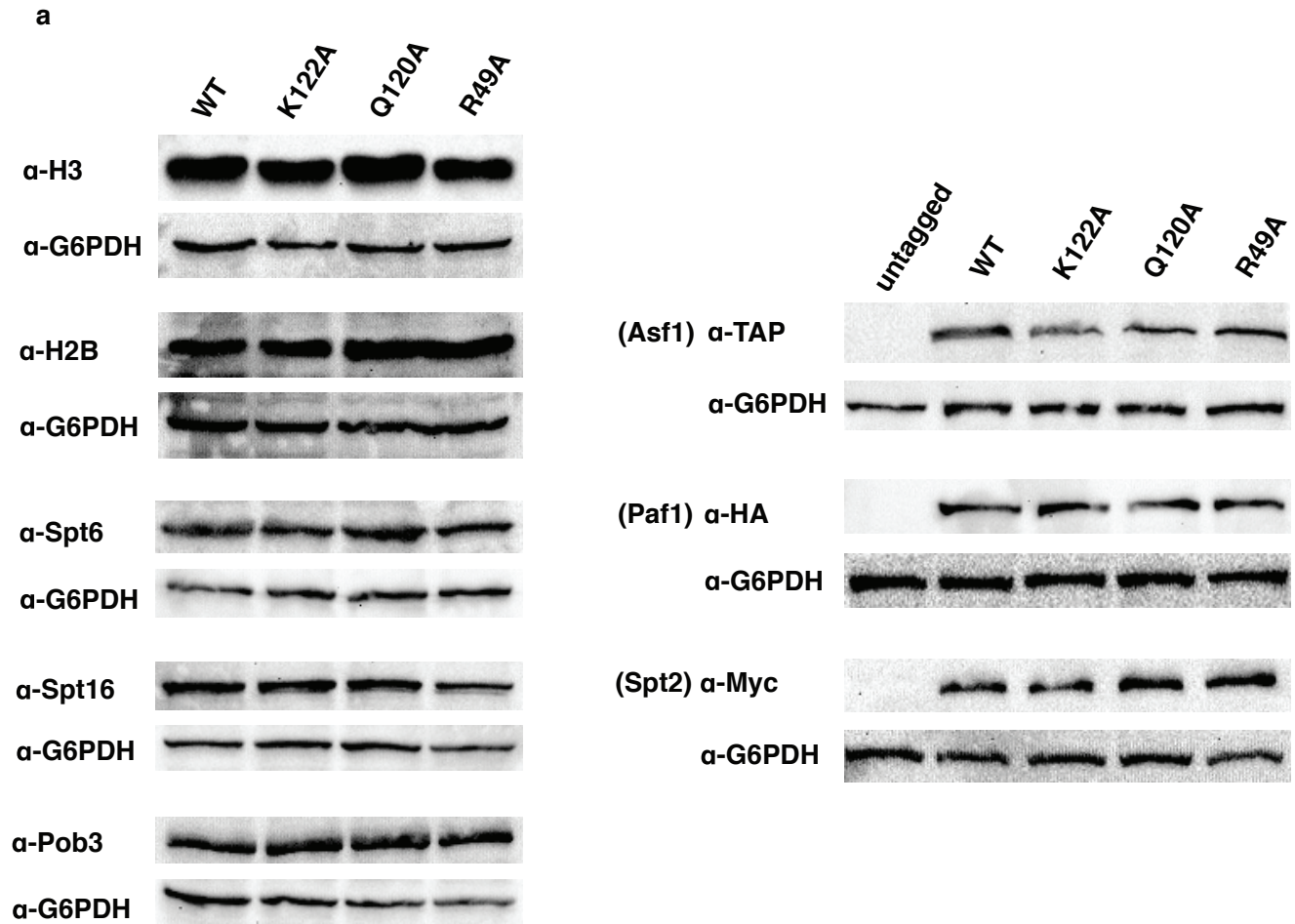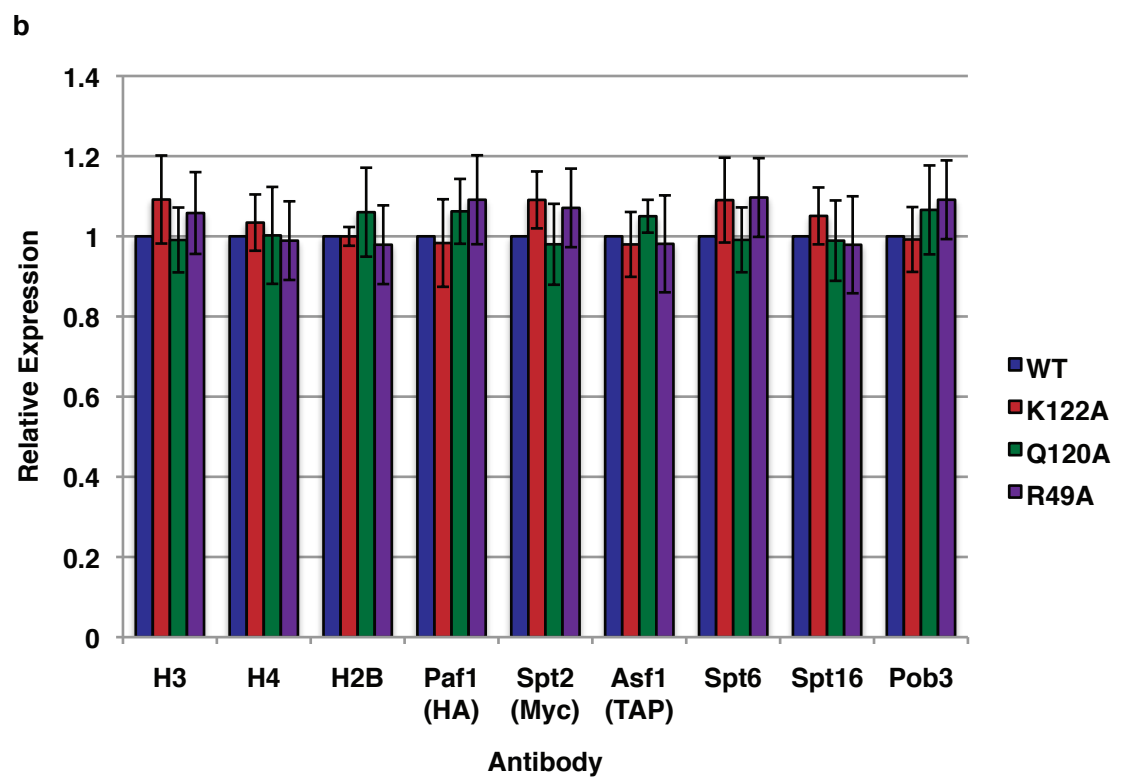

Supplement: Supplementary file 1 — 10.1186/s13072-016-0066-4 Histone residue substitutions do not alter total protein levels. (a) Western analysis examining the effect of histone mutants on total histone H3, H2B, Spt6, Spt16, Pob3, Asf1-TAP, HA-Paf1, and Spt2-Myc protein levels. Strains expressing the indicated histone alleles (YS417, YS404, YS409, YS428, YS454, YS458, YS462, YS471, YS493, YS504, YS518, YS525) were grown to approximately 3 × 10^7^ cells/mL in YPD at 30 °C. Proteins were extracted with trichloroacetic acid and subjected to Western analysis using anti-H3, anti-H2B, anti-Spt6, anti-Spt16, anti-Pob3, anti-PAP, anti-HA, anti-Myc, and anti-G6PDH (loading control). (b) Quantitation of Western analysis, where similar results were obtained for three independent experiments and WT was arbitrarily set to 1 and error bars represent the mean ± SEM of three biological replicate experiments. [file 13072_2016_66_MOESM1_ESM.pdf]

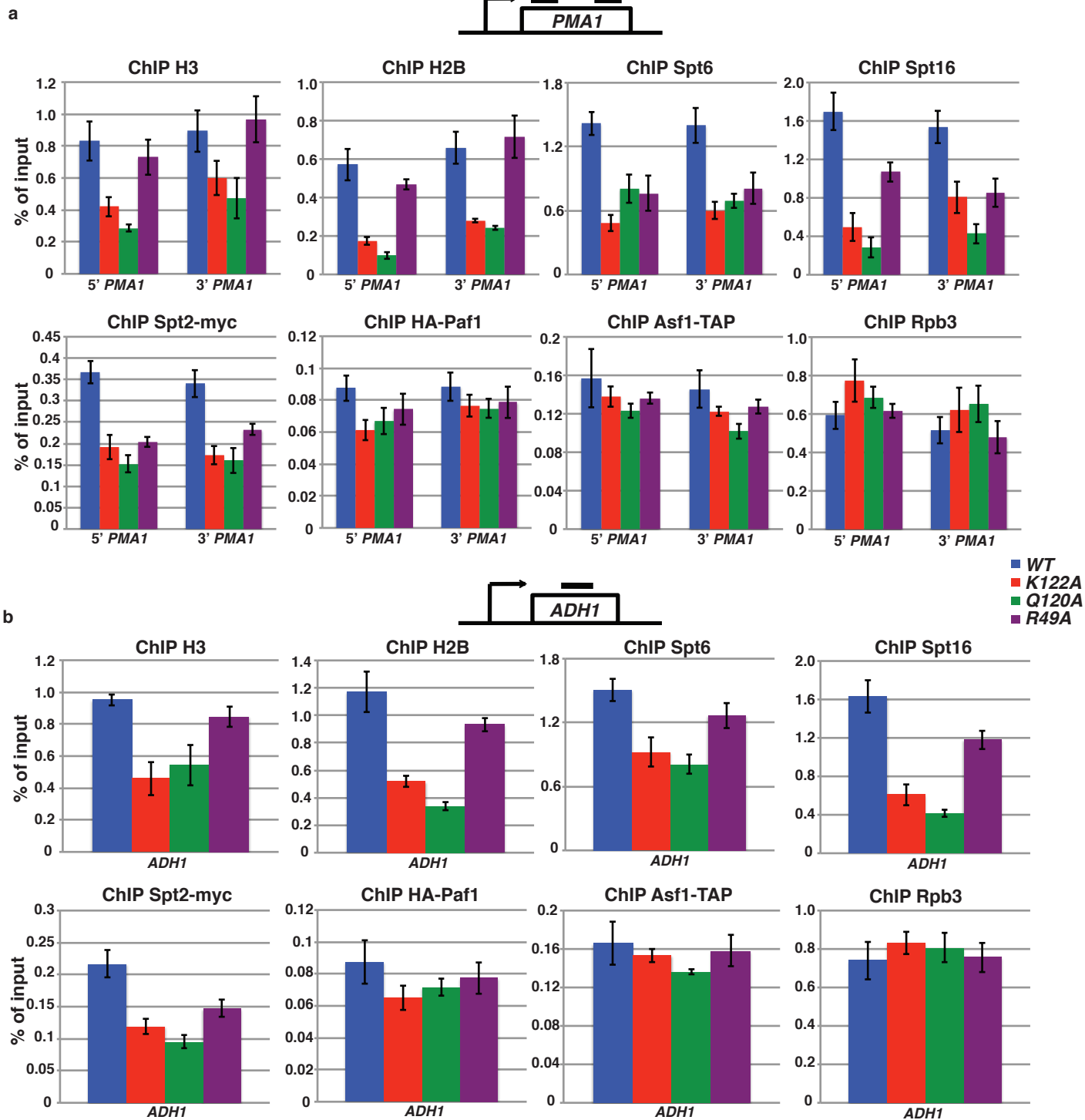

Supplement: Supplementary file 2 — 10.1186/s13072-016-0066-4 Histone mutations result in decreased Spt2, Spt6, and Spt16, occupancy over highly transcribed genes. (a) ChIP analysis was performed on chromatin prepared from strains expressing HHTS-HHFS alleles (YS454-YS456, YS493-YS495) or the indicated histone mutant alleles (YS458-YS462, YS465, YS471, YS472, YS474, YS504-YS506, YS518, YS519, YS521, YS525-YS527) that were grown in YPD at 30 °C. The amount of immunoprecipitated DNA was determined by qPCR and is shown as a percentage of the input material and represents the mean ± SEM of three biological replicate experiments. Factor occupancy was measured within the coding region of a highly transcribed gene, PMA1. The regions assayed by qPCR are marked with the black bars in the diagram provided for the gene. (b) Factor occupancy at ADH1, a highly transcribed gene, was determined as described in a. [file 13072_2016_66_MOESM2_ESM.pdf]

a

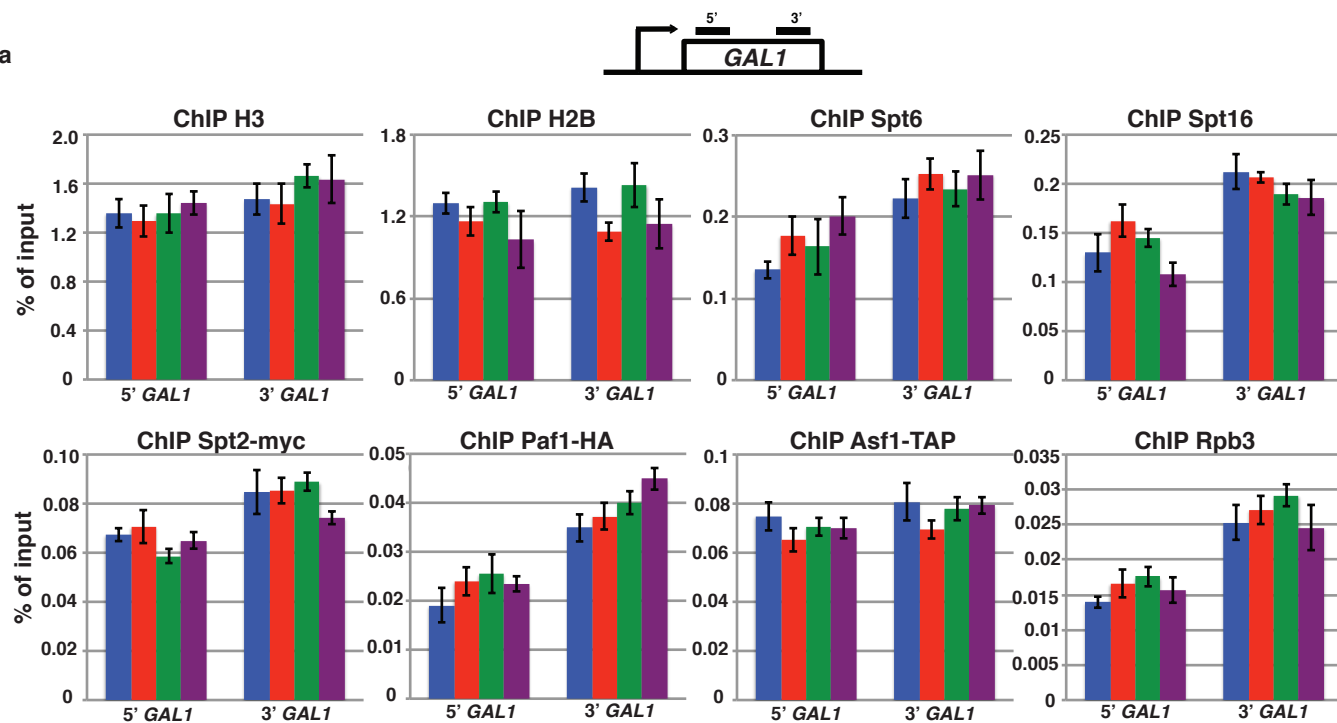

b

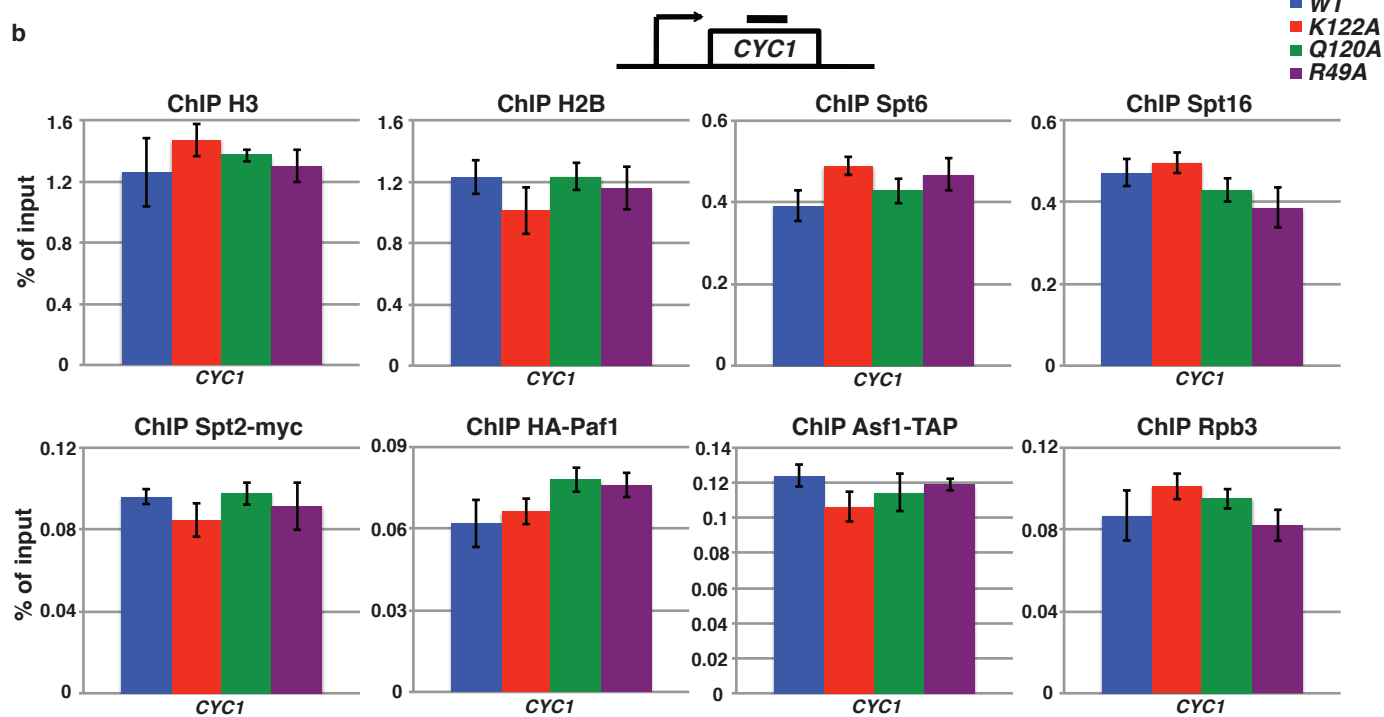

Supplement: Supplementary file 3 — 10.1186/s13072-016-0066-4 Histone mutations do not alter Spt2, Spt6, and Spt16, occupancy over lowly transcribed genes. (a) ChIP analysis was performed on chromatin prepared from strains expressing HHTS-HHFS alleles (YS454-YS456, YS493-YS495) or the indicated histone mutant alleles (YS458-YS462, YS465, YS471, YS472, YS474, YS504-YS506, YS518, YS519, YS521, YS525-YS527) that were grown in YPD at 30 °C. The amount of immunoprecipitated DNA was determined by qPCR and is shown as a percentage of the input material and represents the mean ± SEM of three biological replicate experiments. Factor occupancy was measured within the coding region of a GAL1. (b) Factor occupancy at CYC1, a lowly transcribed gene, was determined as described in a. [file 13072_2016_66_MOESM3_ESM.pdf]

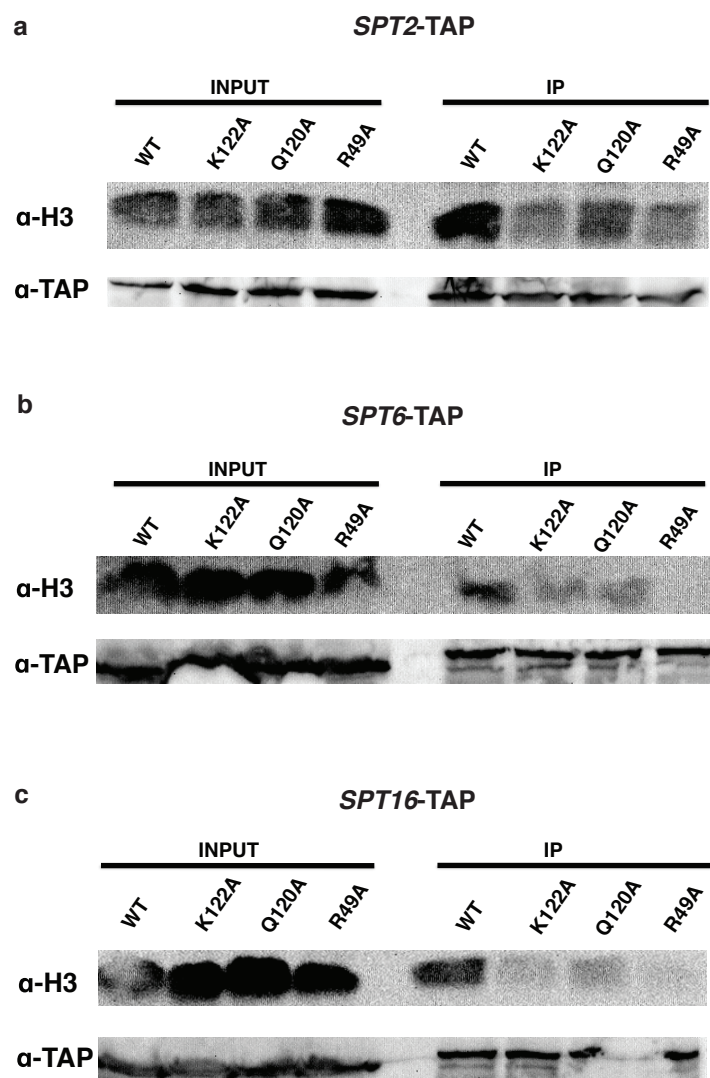

Supplement: Supplementary file 4 — 10.1186/s13072-016-0066-4 Histone mutants cause decreased interaction with Spt2, Spt6, and Spt16. Pull down of Spt2-TAP (a), Spt6-TAP (b), or Spt16-TAP (c) in strains expressing WT, H3 K122A, H3 Q120A, or H3 R49A histone alleles. Extracts from strains expressing WT (YS482, YS485, YS490), K122A (YS497, YS538, YS501), Q120A (YS508, YS511, YS514) or R49A (YS565, YS570, YS522) were incubated with IgG sepharose. Immunoblot analysis was performed to assess the presence of histone H3 and TAP-Spt2, Spt6, or Spt16 in the pull-down fractions (lanes 5–8). Lanes 1–4 in each blot represent 1 % of input material. [file 13072_2016_66_MOESM4_ESM.pdf]
