# Supplementary material for: Regulation of chaperone binding and nucleosome dynamics by key residues within the globular domain of histone H3
Source: Epigenetics Chromatin. 2016 Apr 30;9:17. doi: 10.1186/s13072-016-0066-4 (PMC4851828; doi:10.1186/s13072-016-0066-4)
Supplement: Supplementary file 5 — 10.1186/s13072-016-0066-4 Saccharomyces cerevisiae strains used in this study. [file 13072_2016_66_MOESM5_ESM.docx]

**Additional file 5: Table S1. *Saccharomyces cerevisiae* strains used.**

| **Strain** | **Genotype** | **Reference or**  **Source** |
| --- | --- | --- |
| FY4 | *MAT***a** | (WINSTON *et al.*  1995) |
| FY346 | *MAT***a** *leu2*∆*1 lys2-128*δ *ura3-52 spt16-197* | (MALONE *et al.*  1991) |
| FY2180 | *MAT***a** *his4-912*δ *leu2*∆*1 lys2-128*δ *FLAG-spt6-1004* | (KAPLAN *et al.*  2003) |
| YJ1 | *MAT***a** *ura3*∆*0 trp1*∆*63 lys2*∆*0 met15*∆*0* | This study |
| YJ780 | *MAT***a** *his3*∆*200*, *lys2*-*128*δ, *leu2*∆*1*, *spt2*∆*0*::KanMX | This study |
| YJ926 | *MAT*α *his3*∆*200 leu2*∆*0 trp1*∆*63 ura3*∆*0 (hht1-hhf1)*∆*::NatMX4 (hht2-hhf2)*∆*::hhts-K122A/HHFS-URA3 can1*∆*::MFApr-HIS3* | (HAINER and  MARTENS 2011) |
| YJ927 | *MAT*α *his3*∆*200 leu2*∆*0 lys2*∆*0 ura3*∆*0 met15*∆*0 (hht1- hhf1)*∆*::NatMX4 (hht2-hhf2)*∆*::HHTS/HHFS-URA3 can1*∆*::MFApr- HIS3* | (HAINER and  MARTENS 2011) |
| YJ933 | *MAT*α *his3*∆*200 leu2*∆*0 ura3*∆*0 trp1*∆*63 (hht1-hhf1)*∆*::NatMX4 (hht2-hhf2)*∆*::hhts-Q120A/HHFS-URA3* | (HAINER and  MARTENS 2011) |
| YJ938 | *MAT***a** *his3*∆*200 leu2*∆*0 lys2*∆*0 ura3*∆*0 trp1*∆*63 (hht1- hhf1)*∆*::NatMX4 (hht2-hhf2)*∆*::hhts-R49A/HHFS-URA3* | (HAINER and  MARTENS 2011) |
| YTM194 | *MAT***a** *ura3*∆*0 hht1*∆::*HHTS-URA3* (*hht2-hhf2*)∆::*HHTS/HHFS- URA3* | T. Mavrich |
| YTM202 | *MAT***a** *ura3*∆*0 hht1*∆::*hhts-K122A-URA3* (*hht2-hhf2*)∆::*hhts- K122A/HHFS-URA3* | T. Mavrich |
| YS288 | *MAT***a** *his3*Δ*200 leu2*Δ*0 ura3*Δ*0* (or -*52*) *lys2*-*128*δ *met15*Δ*0*  *can1*Δ::*MFA1*pr-*HIS3 snf2*Δ::*LEU2 hht1*Δ::*URA3*-*hhts*(*K122A*), (*hht2*-*hhf2*)Δ::*URA3*-*hhts*(*K122A*)-*HHFS* | This study |
| YS289 | *MAT*α *his3*Δ*200 leu2*Δ*0 ura3*Δ*0* (or -*52*) *lys2*-*128*δ *met15*Δ*0 can1*Δ::*MFA1*pr-*HIS3 snf2*Δ::*LEU2 hht1*Δ::*URA3*-*hhts*(*K122A*), (*hht2*-*hhf2*)Δ::*URA3*-*hhts*(*K122A*)-*HHFS* | This study |
| YS380 | *MAT***a** *ura3*∆*0 leu2*∆*0 his3*∆*200 lys2*∆*0 met15*∆*0 snf2*∆::*LEU2* (*hht1*-  *hhf1*)∆::*hhts-K122A*/*HHFS*-*URA3* (*hht2*-*hhf2*)∆::*hhts-K122A*/*HHFS*-  *URA3* | This study |
| YS381 | *MAT***a** *ura3*∆*0 leu2*∆*0 lys2*∆*0 met15*∆*0 snf2*∆::*LEU2 can1*∆::*MFA1*pr-  *HIS3* (*hht1*-*hhf1*)∆::*HHTS/HHFS*-*URA3* (*hht2*-*hhf2*)∆::*HHTS/HHFS*-  *URA3* | This study |
| YS393 | *MAT*α *his3*Δ*200 leu2*Δ*0 ura3*Δ*0* (or -*52*) *lys2*-*128*δ *snf2*Δ::*LEU2*  *srg1*-*1 hht1*Δ::*URA3*-*hhts*(*K122A*) (*hht2*-*hhf2*)Δ::*URA3*-*hhts*(*K122A*)- *hht1*Δ::*URA3*-*hhts*(*K122A*) (*hht2*-*hhf2*)Δ::*URA3*-*hhts*-*K122A*/*HHFS*  *HHFS* | This study |
| YS399 | *MAT***a** *his3*Δ*200 leu2*Δ*0 ura3*Δ*0* (or -*52*) *lys2*-*128*δ *snf2*Δ::*LEU2 srg1*-*1 hht1*Δ::*URA3*-*HHTS* (*hht2*-*hhf2*)Δ::*HHTS/HHFS-URA3* | This study |
| YS400 | *MAT*α *his3*Δ*200 leu2*Δ*0 ura3*Δ*0* (or -*52*) *lys2-128*δ *srg1-1 hht1*Δ::*URA3*-*HHTS* (*hht2*-*hhf2*)Δ::*HHTS/HHFS-URA3* | This study |
| YS404 | *MAT***a** *his3*∆*200 leu2*∆*0 lys2*∆*0 trp1*∆*63 ura3*∆*0 met15*∆*0* (*hht1*-  *hhf1*)∆::*hhts-K122A*/*HHFS*-Hygro (*hht2*-*hhf2*)∆::*hhts-K122A*/*HHFS*-  *URA3 can1*∆::*MFA*pr-*HIS3* | This study |
| YS405 | *MAT***a** *his3*∆*200 leu2*∆*0 lys2*∆*0 trp1*∆*63 ura3*∆*0 met15*∆*0* (*hht1*-  *hhf1*)∆::*hhts-K122R*/*HHFS*-Hygro (*hht2*-*hhf2*)∆::*hhts-K122R*/*HHFS*-  *URA3 can1*∆::*MFA*pr-*HIS3* | This study |
| YS407 | *MAT***a** *his3*∆*200 leu2*∆*0 lys2*∆*0 trp1*∆*63 ura3*∆*0 met15*∆*0* (*hht1*-  *hhf1*)∆::*hhts-K122Q*/*HHFS*-Hygro (*hht2*-*hhf2*)∆::*hhts-K122Q*/*HHFS*-  *URA3 can1*∆::*MFA*pr-*HIS3* | This study |
| YS409 | *MAT***a** *his3*∆*200 leu2*∆*0 lys2*∆*0 trp1*∆*63 ura3*∆*0 met15*∆*0* (*hht1*-  *hhf1*)∆::*hhts-Q120A*/*HHFS*-Hygro (*hht2*-*hhf2*)∆::*hhts-Q120A*/*HHFS*-  *URA3 can1*∆::*MFA*pr-*HIS3* | This study |
| YS411 | *MAT***a** *his3*∆*200 leu2*∆*0 lys2*∆*0 trp1*∆*63 ura3*∆*0 met15*∆*0* (*hht1*- *hhf1*)∆::*hhts-V117A*/*HHFS*-Hygro (*hht2*-*hhf2*)∆::*hhts-V117A*/*HHFS*-  *URA3 can1*∆::*MFA*pr-*HIS3* | This study |
| YS413 | *MAT***a** *his3*∆*200 leu2*∆*0 lys2*∆*0 trp1*∆*63 ura3*∆*0 met15*∆*0* (*hht1*- *hhf1*)∆::*hhts-V46A*/*HHFS*-Hygro (*hht2*-*hhf2*)∆::*hhts-V46A*/*HHFS*- *URA3 can1*∆::*MFA*pr-*HIS3* | This study |
| YS415 | *MAT***a** *his3*∆*200 leu2*∆*0 lys2*∆*0 trp1*∆*63 ura3*∆*0 met15*∆*0* (*hht1*- *hhf1*)∆::*HHTS*/*hhfs-I46A*-Hygro (*hht2*-*hhf2*)∆::*HHTS*/*hhfs-I46A*- *URA3 can1*∆::*MFA*pr-*HIS3* | This study |
| YS417 | *MAT***a** *his3*∆*200 leu2*∆*0 lys2*∆*0 trp1*∆*63 ura3*∆*0 met15*∆*0* (*hht1*-  *hhf1*)∆::*HHTS*/*HHFS*-Hygro (*hht2*-*hhf2*)∆::*HHTS*/*HHFS*-*URA3 can1*∆::*MFA*pr-*HIS3* | This study |
| YS428 | *MAT***a** *his3*∆*200 leu2*∆*0 lys2*∆*0 trp1*∆*63 ura3*∆*0 met15*∆*0* (*hht1*-  *hhf1*)∆::*hhts-R49A*/*HHFS*-Hygro (*hht2*-*hhf2*)∆::*hhts-R49A*/*HHFS*-  *URA3 can1*∆::*MFA*pr-*HIS3* | This study |
| YS454 | *MAT*α *his3*∆*200 ura3*∆*0 lys2*∆*0 met15*∆*0* (*hht1*-  *hhf1*)∆::*HHTS/HHFS*-Hygro (*hht2*-*hhf2*)∆::*HHTS/HHFS*-*URA3*  *SPT2*-13Myc::KanMX HA-*PAF1* | This study |
| YS455 | *MAT*α *his3*∆*200 ura3*∆*0 lys2*∆*0* (*hht1*-*hhf1*)∆::*HHTS/HHFS*-Hygro  (*hht2*-*hhf2*)∆::*HHTS/HHFS*-*URA3 SPT2*-13Myc::KanMX HA-*PAF1* | This study |
| YS456 | *MAT***a** *his3*∆*200 ura3*∆*0 leu2*∆*0 lys2*∆*0 met15*∆*0* (*hht1*-  *hhf1*)∆::*HHTS/HHFS*-Hygro (*hht2*-*hhf2*)∆::*HHTS/HHFS*-*URA3*  *SPT2*-13Myc::KanMX HA-*PAF1* | This study |
| YS458 | *MAT*α *his3*∆*200 ura3*∆*0 leu2*∆*0 trp1*∆*63* (*hht1*-*hhf1*)∆::*hhts-K122A*/*HHFS*-Hygro (*hht2*-*hhf2*)∆::*hhts-K122A*/*HHFS*-*URA3 SPT2*-  13Myc::KanMX HA-*PAF1* | This study |
| YS459 | *MAT*α *his3*∆*200 ura3*∆*0 lys2*∆*0* (*hht1*-*hhf1*)∆::*hhts-K122A*/*HHFS*- Hygro (*hht2*-*hhf2*)∆::*hhts-K122A*/*HHFS*-*URA3 SPT2*-  13Myc::KanMX HA-*PAF1* | This study |
| YS460 | *MAT***a** *his3*∆*200 ura3*∆*0 leu2*∆*0 met15*∆*0* (*hht1*-*hhf1*)∆::*hhts- K122A*/*HHFS*-Hygro (*hht2*-*hhf2*)∆::*hhts-K122A*/*HHFS*-*URA3 SPT2*-  13Myc::KanMX HA-*PAF1* | This study |
| YS462 | *MAT***a** *his3*∆*200 ura3*∆*0 leu2*∆*0 lys2*∆*0* (*hht1*-*hhf1*)∆::*hhts- Q120A*/*HHFS*-Hygro (*hht2*-*hhf2*)∆::*hhts-Q120A*/*HHFS*-*URA3 SPT2*-  13Myc::KanMX HA-*PAF1* | This study |
| YS463 | *MAT*α *his3*∆*200 ura3*∆*0 trp1*∆*63 lys2*∆*0* (*hht1*-*hhf1*)∆::*hhts- Q120A*/*HHFS*-Hygro (*hht2*-*hhf2*)∆::*hhts-Q120A*/*HHFS*-*URA3 SPT2*-  13Myc::KanMX HA-*PAF1* | This study |
| YS465 | *MAT***a** *his3*∆*200 ura3*∆*0 leu2*∆*0* (*hht1*-*hhf1*)∆::*hhts-Q120A*/*HHFS*- Hygro (*hht2*-*hhf2*)∆::*hhts-Q120A*/*HHFS*-*URA3 SPT2*-  13Myc::KanMX HA-*PAF1* | This study |
| YS471 | *MAT***a** *his3*∆*200 ura3*∆*0 leu2*∆*0 lys2*∆*0 trp1*∆*63* (*hht1*-*hhf1*)∆::*hhts- R49A*/*HHFS*-Hygro (*hht2*-*hhf2*)∆::*hhts-R49A*/*HHFS*-*URA3 SPT2*-  13Myc::KanMX HA-*PAF1* | This study |
| YS472 | *MAT***a** *his3*∆*200 ura3*∆*0 leu2*∆*0 lys2*∆*0 trp1*∆*63 met15*∆*0* (*hht1*-  *hhf1*)∆::*hhts-R49A*/*HHFS*-Hygro (*hht2*-*hhf2*)∆::*hhts-R49A*/*HHFS*-  *URA3 SPT2*-13Myc::KanMX HA-*PAF1* | This study |
| YS474 | *MAT*α *his3*∆*200 ura3*∆*0 leu2*∆*0 lys2*∆*0* (*hht1*-*hhf1*)∆::*hhts- R49A*/*HHFS*-Hygro (*hht2*-*hhf2*)∆::*hhts-R49A*-*URA3 SPT2*-13Myc::KanMX HA-*PAF1* | This study |
| YS482 | *MAT***a** *his3*∆*200 met15*∆*0 ura3*∆*0 lys2*∆*0* (*hht1*-  *hhf1*)∆::*HHTS/HHFS*-Hygro (*hht2*-*hhf2*)∆::*HHTS/HHFS*-*URA3*  *SPT2*-TAP::*HIS3* HA-*PAF1* | This study |
| YS485 | *MAT***a** *his3*∆*200 met15*∆*0 ura3*∆*0 lys2*∆*0 leu2*∆*0* (*hht1*-  *hhf1*)∆::*HHTS/HHFS*-Hygro (*hht2*-*hhf2*)∆::*HHTS/HHFS*-*URA3* *SPT2*-13myc::KanMX HA-*PAF1 SPT6*-TAP::*HIS3* | This study |
| YS475 | *MAT***a** *met15*Δ*0* *ura3*Δ*0* *leu2*Δ*0* *lys2*Δ*0*, (*hht1*-*hhf1*)Δ::*HHTS/HHFS*-Hygro (*hht2*-*hhf2*)Δ::*HHTS/HHFS*-*URA3* *SPT2*-13Myc::*KanMX* HA-*PAF1* *GAL1*pr-*FMP27*::*KanMX4* | This study |
| YS477 | *MAT*α *met15*Δ*0* *ura3*Δ*0* *leu2*Δ*0* (*hht1*-*hhf1*)Δ::*HHTS/HHFS*-Hygro (*hht2*-*hhf2*)Δ::*HHTS/HHFS*-*URA3* *SPT2*-13Myc::*KanMX*, HA-*PAF1* *GAL1*pr-*FMP27*::*KanMX4* | This study |
| YS478 | *MAT***a** *met15*Δ*0* *ura3*Δ*0* *leu2*Δ*0* *his3*∆*200* (*hht1*-*hhf1*)Δ::*HHTS/HHFS*-Hygro (*hht2*-*hhf2*)Δ::*HHTS/HHFS*-*URA3* *SPT2*-13Myc::*KanMX* HA-*PAF1* *GAL1*pr-*FMP27*::*KanMX4* | This study |
| YS490 | *MAT***a** *his3*∆*200 met15*∆*0 ura3*∆*0 lys2*∆*0 leu2*∆*0* (*hht1*-  *hhf1*)∆::*HHTS/HHFS*-Hygro (*hht2*-*hhf2*)∆::*HHTS/HHFS*-*URA3* *SPT2*-13myc::KanMX HA-*PAF1 SPT16*-TAP::*HIS3* | This study |
| YS493 | *MAT***a** *his3*∆*200 met15*∆*0 ura3*∆*0* (*hht1*-*hhf1*)∆::*HHTS/HHFS*-Hygro  (*hht2*-*hhf2*)∆::*HHTS/HHFS*-*URA3 SPT2*-13myc::KanMX *ASF1*-TAP::*HIS3* | This study |
| YS494 | *MAT*α *his3*∆*200 met15*∆*0 ura3*∆*0* (*hht1*-*hhf1*)∆::*HHTS/HHFS*-Hygro  (*hht2*-*hhf2*)∆::*HHTS/HHFS*-*URA3 SPT2*-13myc::KanMX *ASF1*- TAP::*HIS3* HA-*PAF1* | This study |
| YS495 | *MAT*α *his3*∆*200 met15*∆*0 ura3*∆*0* (*hht1*-*hhf1*)∆::*HHTS/HHFS*-Hygro  (*hht2*-*hhf2*)∆::*HHTS/HHFS*-*URA3 SPT2*-13myc::KanMX *ASF1*- TAP::*HIS3* HA-*PAF1* | This study |
| YS497 | *MAT***a** *his3*∆*200 met15*∆*0 ura3*∆*0 trp1*∆*63 leu2*∆0 (*hht1*-*hhf1*)∆::*hhts- K122A*/*HHFS*-Hygro (*hht2*-*hhf2*)∆::*hhts-K122A*/*HHFS*-*URA3 SPT2*-  TAP::*HIS3* HA-*PAF1* | This study |
| YS501 | *MAT***a** *his3*∆*200 met15*∆*0 ura3*∆*0 trp1*∆*63 leu2*∆*0* (*hht1*-*hhf1*)∆::*hhts- K122A*/*HHFS*-Hygro (*hht2*-*hhf2*)∆::*hhts-K122A*/*HHFS*-*URA3 SPT2*-  13myc::KanMX *SPT16*-TAP::*HIS3* | This study |
| YS504 | *MAT***a** *his3*∆*200 met15*∆*0 ura3*∆*0 trp1*∆*63 leu2*∆*0* (*hht1*-*hhf1*)∆::*hhts- K122A*/*HHFS*-Hygro (*hht2*-*hhf2*)∆::*hhts-K122A*/*HHFS*-*URA3 SPT2*-  13myc::KanMX *ASF1*-TAP::*HIS3* HA-*PAF1* | This study |
| YS505 | *MAT*α *his3*∆*200 met15*∆*0 ura3*∆*0 leu2*∆*0* (*hht1*-*hhf1*)∆::*hhts- K122A*/*HHFS*-Hygro (*hht2*-*hhf2*)∆::*hhts-K122A*/*HHFS*-*URA3 SPT2*-  13myc::KanMX *ASF1*-TAP::*HIS3* HA-*PAF1* | This study |
| YS506 | *MAT***a** *his3*∆*200 met15*∆*0 ura3*∆*0 trp1*∆*63 leu2*∆*0* (*hht1*-*hhf1*)∆::*hhts- K122A*/*HHFS*-Hygro (*hht2*-*hhf2*)∆::*hhts-K122A/HHFS*-*URA3 SPT2*-  13myc::KanMX *ASF1*-TAP::*HIS3* HA-*PAF1* | This study |
| YS508 | *MAT*α *his3*∆*200 met15*∆*0 ura3*∆*0 trp1*∆*63 leu2*∆*0 lys2*∆*0* (*hht1*-  *hhf1*)∆::*hhts-Q120A/HHFS*-Hygro (*hht2*-*hhf2*)∆::*hhts-Q120A*/*HHFS*-  *URA3 SPT2*-TAP::*HIS3* HA-*PAF1* | This study |
| YS511 | *MAT***a** *his3*∆*200 met15*∆*0 ura3*∆*0 trp1*∆*63 lys2*∆*0* (*hht1*-  *hhf1*)∆::*hhts-Q120A*/*HHFS*-Hygro (*hht2*-*hhf2*)∆::*hhts-Q120A*/*HHFS*-  *URA3 SPT2*-13myc::KanMX *SPT6*-TAP::*HIS3* | This study |
| YS514 | *MAT*α *his3*∆*200 met15*∆*0 ura3*∆*0 lys2*∆*0* (*hht1*-*hhf1*)∆::*hhts- Q120A*/*HHFS*-Hygro (*hht2*-*hhf2*)∆::*hhts-Q120A*/*HHFS*-*URA3 SPT2*-  13myc::KanMX *SPT16*-TAP::*HIS3* HA-*PAF1* | This study |
| YS518 | *MAT***a** *his3*∆*200 ura3*∆*0 lys2*∆*0 leu2*∆*0* (*hht1*-*hhf1*)∆::*hhts- Q120A*/*HHFS*-Hygro (*hht2*-*hhf2*)∆::*hhts-Q120A*/*HHFS*-*URA3 SPT2*-  13myc::KanMX *ASF1*-TAP::*HIS3* HA-*PAF1* | This study |
| YS519 | *MAT*α *his3*∆*200 ura3*∆*0 met15*∆*0 trp1*∆*63 leu2*∆*0* (*hht1*-  *hhf1*)∆::*hhts-Q120A*/*HHFS*-Hygro (*hht2*-*hhf2*)∆::*hhts-Q120A*/*HHFS*-  *URA3 SPT2*-13myc::KanMX *ASF1*-TAP::*HIS3* HA-*PAF1* | This study |
| YS521 | *MAT***a** *his3*∆*200 ura3*∆*0 met15*∆*0 trp1*∆*63 leu2*∆*0* (*hht1*-*hhf1*)∆::*hhts- Q120A*/*HHFS*-Hygro (*hht2*-*hhf2*)∆::*hhts-Q120A*/*HHFS*-*URA3 SPT2*-  13myc::KanMX *ASF1*-TAP::*HIS3* | This study |
| YS522 | *MAT***a** *his3*∆*200 ura3*∆*0 met15*∆*0 lys2*∆*0 leu2*∆*0* (*hht1*-*hhf1*)∆::*hhts- R49A*/*HHFS*-Hygro (*hht2*-*hhf2*)∆::*hhts-R49A*/*HHFS*-*URA3 SPT2*-  13myc::KanMX *SPT16*-TAP::*HIS3* | This study |
| YS525 | *MAT*α *his3*∆*200 ura3*∆*0 met15*∆*0 leu2*∆*0* (*hht1*-*hhf1*)∆::*hhts- R49A*/*HHFS*-Hygro (*hht2*-*hhf2*)∆::*hhts-R49A*/*HHFS*-*URA3 SPT2*-  13myc::KanMX *ASF1*-TAP::*HIS3* HA-*PAF1* | This study |
| YS526 | *MAT*α *his3*∆*200 ura3*∆*0 leu2*∆*0* (*hht1*-*hhf1*)∆::*hhts-R49A*/*HHFS*- Hygro (*hht2*-*hhf2*)∆::*hhts-R49A*/*HHFS*-*URA3 SPT2*-13myc::KanMX  *ASF1*-TAP::*HIS3* | This study |
| YS527 | *MAT*α *his3*∆*200 ura3*∆*0 leu2*∆*0* (*hht1*-*hhf1*)∆::*hhts-R49A*/*HHFS*- Hygro (*hht2*-*hhf2*)∆::*hhts-R49A*/*HHFS*-*URA3 SPT2*-13myc::KanMX  *ASF1*-TAP::*HIS3* | This study |
| YS538 | *MAT*α *ura3*∆*0 leu2*∆*0* (*hht1*-*hhf1*)∆::*hhts-K122A*/*HHFS*-Hygro (*hht2*-  *hhf2*)∆::*hhts-K122A*/*HHFS*-*URA3 SPT2*-13myc::KanMX *SPT6*- TAP::*HIS3* HA-*PAF1* | This study |
| YS541 | *MAT***a** *his3*∆*200 met15*∆*0 ura3*∆*0 lys2*∆*0 leu2*∆*0 HHT1*-*HHF1* (*hht2*-  *hhf2*)∆::*HHTS/HHFS*-*URA3 SPT2*-TAP::*HIS3* | This study |
| YS542 | *MAT***a** *his3*∆*200 met15*∆*0 ura3*∆*0* *leu2*∆*0 HHT1*-*HHF1* (*hht2*-  *hhf2*)∆::*HHTS/HHFS*-*URA3* *SPT2*-13myc::KanMX | This study |
| YS544 | *MAT*α *his3*∆*200 met15*∆*0 ura3*∆*0* (*hht1*-*hhf1*)∆::*HHTS/HHFS*-Hygro  *HHT2*-*HHF2 SPT2*-TAP::*HIS3* | This study |
| YS545 | *MAT*α *his3*∆*200 met15*∆*0 ura3*∆0 *lys2*∆*0* (*hht1*-*hhf1*)∆::*HHTS/HHFS*-Hygro *HHT2*-*HHF2* *SPT2*-13myc::KanMX | This study |
| YS547 | *MAT***a** *his3*∆*200 met15*∆*0 ura3*∆*0 leu2*∆*0 HHT1*-*HHF1* (*hht2*-  *hhf2*)∆::*hhts*-*K122A*/*HHFS*-*URA3 SPT2*-TAP::*HIS3* | This study |
| YS548 | *MAT***a** *his3*∆*200 lys2*∆*0 ura3*∆0 *leu2*∆*0 trp1*∆*63 HHT1*-*HHF1* *hht2*-  *hhf2*)∆::*hhts*-*K122A*/*HHFS*-*URA3 SPT2*-13myc::KanMX | This study |
| YS550 | *MAT*α *his3*∆*200 met15*∆*0 ura3*∆*0 leu2*∆*0 trp1*∆*63* (*hht1*-  *hhf1*)∆::*hhts*-*K122A*/*HHFS*-Hygro *HHT2*-*HHF2 SPT2*-TAP::*HIS3* | This study |
| YS551 | *MAT*α *his3*∆*200 met15*∆*0 ura3*∆*0 leu2*∆*0* (*hht1*-  *hhf1*)∆::*hhts*-*K122A*/*HHFS*-Hygro *HHT2*-*HHF2 SPT2*-TAP::*HIS3* | This study |
| YS553 | *MAT***a** *his3*∆*200 met15*∆*0 lys2*∆*0 trp1*∆*63 ura3*∆*0 leu2*∆*0 HHT1*-  *HHF1* (*hht2*-*hhf2*)∆::*hhts-Q120A*/*HHFS*-*URA3 SPT2*-myc::KanMX | This study |
| YS554 | *MAT***a** *his3*∆*200 met15*∆*0 lys2*∆*0 trp1*∆*63 ura3*∆*0 leu2*∆*0 HHT1*-  *HHF1* (*hht2*-*hhf2*)∆::*hhts-Q120A*/*HHFS*-*URA3* *SPT2*-TAP::*HIS3* | This study |
| YS557 | *MAT*α *his3*∆*200 met15*∆*0 ura3*∆*0* (*hht1*-*hhf1*)∆::*hhts-Q120A*/*HHFS*-Hygro *HHT2*-*HHF2 SPT2*-myc::KanMX | This study |
| YS558 | *MAT***a** *his3*∆*200 met15*∆*0 ura3*∆*0 lys2*∆*0 trp1*∆*63* (*hht1*-*hhf1*)∆::*hhts-Q120A*/*HHFS*-Hygro *HHT2*-*HHF2 SPT2*-TAP::*HIS3* | This study |
| YS560 | *MAT***a** *his3*∆*200 met15*∆*0 leu2*∆*0 ura3*∆*0 HHT1*-*HHF1* (*hht2*-  *hhf2*)∆::*hhts-R49A*/*HHFS*-*URA3 SPT16*-TAP::*HIS3 SPT2*-myc::KanMX | This study |
| YS563 | *MAT***a** *his3*∆*200 leu2*∆*0 ura3*∆*0 met15*∆*0* (*hht1*-*hhf1*)∆::*hhts- R49A/HHFS*-Hygro *HHT2*-*HHF2* | This study |
| YS565 | *MAT***a** *his3*∆*200 ura3*∆*0 met15*∆*0 leu2*∆*0 lys2*∆*0* (*hht1*-*hhf1*)∆::*hhts- R49A*/*HHFS*-Hygro (*hht2*-*hhf2*)∆::*hhts-R49A*/*HHFS*-*URA3 SPT2*-  TAP::*HIS3* | This study |
| YS570 | *MAT***a** *his3*∆*200 ura3*∆*0 leu2*∆*0* (*hht1*-*hhf1*)∆::*hhts-R49A*/*HHFS*- Hygro (*hht2*-*hhf2*)∆::*hhfs-R49A*/*HHFS*-*URA3 SPT2*-myc::KanMX  *SPT6*-TAP::*HIS3* | This study |
| YS585 | *MAT***a** *ura3*Δ*0* *leu2*Δ*0* (*hht1-hhf1*)Δ:: *hhts-K122A*/*HHFS*-Hygro (*hht2-hhf2*)Δ::*hhts-K122A/HHFS*-*URA3* *SPT2*-13Myc::*KanMX* HA-*PAF1* *GAL1*pr-*FMP27*::*KanMX4* | This study |
| YS586 | *MAT***a** *ura3*Δ*0* *leu2*Δ*0* *met15*∆*0 his3*∆*200* (*hht1-hhf1*)Δ:: *hhts-K122A*/*HHFS*-Hygro (*hht2-hhf2*)Δ::*hhts-K122A/HHFS*-*URA3* *SPT2*-13Myc::*KanMX* HA-*PAF1* *GAL1*pr-*FMP27*::*KanMX4* | This study |
| YS587 | *MAT*α *ura3*Δ*0* *his3*∆*200* (*hht1-hhf1*)Δ:: *hhts-K122A*/*HHFS*-Hygro (*hht2-hhf2*)Δ::*hhts-K122A/HHFS*-*URA3* *SPT2*-13Myc::*KanMX* HA-*PAF1* *GAL1*pr-*FMP27*::*KanMX4* | This study |
| YS591 | *MAT*α *ura3*∆*0*, *leu2*∆*0*, *lys2*∆*0*, *his3*∆*200*, (*hht1*-  *hhf1*)∆::*HHTS/HHFS*-Hygro, (*hht2*-*hhf2*)∆::*HHTS/HHFS*-*URA3*, FLAG-*spt6-1004* | This study |
| YS592 | *MAT***a** *ura3*∆*0*, *leu2*∆*0*, *his3*∆*200*, (*hht1*-*hhf1*)∆::*HHTS/HHFS*-Hygro, (*hht2*-*hhf2*)∆::*HHTS/HHFS*-*URA3*, FLAG-*spt6*-*1004* | This study |
| YS593 | *MAT*α *ura3*∆*0*, *leu2*∆*0*, *lys2*∆*0*, *his3*∆*200*, (*hht1*-  *hhf1*)∆::*HHTS/HHFS*-Hygro, (*hht2*-*hhf2*)∆::*HHTS/HHFS*-*URA3*, FLAG-*spt6*-*1004*, *RPB1*-C13Myc::KanMX | This study |
| YS594 | *MAT*α *ura3*∆*0*, *leu2*∆*0*, *lys2*∆*0*, *his3*∆*200*, *met15*∆*0*, (*hht1*-  *hhf1*)∆::*hhts-K122A/HHFS*-Hygro, (*hht2*-*hhf2*)∆::*hhts-K122A/HHFS*-  *URA3*, FLAG-*spt6-1004* | This study |
| YS595 | *MAT*α *ura3*∆*0*, *leu2*∆*0*, *lys2*∆*0*, *his3*∆*200*, (*hht1*-*hhf1*)∆::*hhts- K122A/HHFS*-Hygro, (*hht2*-*hhf2*)∆::*hhts-K122A/HHFS*-*URA3*,  FLAG-*spt6-1004*, *RPB1*-C13Myc::KanMX | This study |
| YS596 | *MAT*α *ura3*∆*0*, *leu2*∆*0*, *lys2*∆*0*, *his3*∆*200*, (*hht1*-*hhf1*)∆::*hhts- K122A*/*HHFS*-Hygro, (*hht2*-*hhf2*)∆::*hhts-K122A*/*HHFS*-*URA3*,  FLAG-*spt6-1004* | This study |
| YS597 | *MAT*α *ura3*∆*0*, *leu2*∆*0*, *lys2*∆*0*, *his3*∆*200*, (*hht1*-*hhf1*)∆::*hhts- Q120A*/*HHFS*-Hygro, (*hht2*-*hhf2*)∆::*hhts-Q120A*/*HHFS*-*URA3*,  FLAG-*spt6-1004* | This study |
| YS598 | *MAT***a** *ura3*∆*0*, *leu2*∆*0*, *lys2*∆*0*, *his3*∆*200*, *trp1*∆*63*, (*hht1*-  *hhf1*)∆::*hhts-Q120A*/*HHFS*-Hygro, (*hht2*-*hhf2*)∆::*hhts-Q120A*/*HHFS*- *URA3*, FLAG-*spt6-1004*, *RPB1*-C13Myc::KanMX, *can1*∆::*MFA*pr- *HIS3* | This study |
| YS599 | *MAT***a** *leu2*∆*0*, *lys2*∆*0*, *his3*∆*200*, (*hht1*-*hhf1*)∆::*hhts-Q120A*/*HHFS*- Hygro, (*hht2*-*hhf2*)∆::*hhts-Q120A*/*HHFS*-*URA3*, FLAG-*spt6-1004*, *can1*∆::*MFA*pr-*HIS3* | This study |
| YS600 | *MAT*α *ura3*∆*0*, *leu2*∆*0*, *lys2*∆*0*, *his3*∆*200*, *met15*∆*0*, *trp1*∆*63* (*hht1*-  *hhf1*)∆::*hhts-R49A*/*HHFS*-Hygro, (*hht2*-*hhf2*)∆::*hhts-R49A*/*HHFS*-  *URA3*, FLAG-*spt6-1004* | This study |
| YS601 | *MAT*α *ura3*∆*0*, *leu2*∆*0*, (*hht1*-*hhf1*)∆::*HHTS/HHFS*-Hygro, (*hht2*-  *hhf2*)∆::*HHTS/HHFS*-*URA3*, *spt16*-*197*, *SPT2*-13Myc::KanMX | This study |
| YS602 | *MAT*α *ura3*∆*0*, *leu2*∆*0*, *his3*∆*200*, (*hht1*-*hhf1*)∆::*HHTS/HHFS*-Hygro,  (*hht2*-*hhf2*)∆::*HHTS/HHFS*-*URA3*, *spt16-197* | This study |
| YS603 | *MAT***a** *ura3*∆*0*, *leu2*∆*0*, *his3*∆*200*, (*hht1*-*hhf1*)∆::*HHTS/HHFS*-Hygro, (*hht2*-*hhf2*)∆::*HHTS/HHFS*-*URA3*, *spt16-197* | This study |
| YS604 | *MAT***a** *ura3*∆*0*, *leu2*∆*0*, *lys2*∆*0*, *trp1*∆*63*, (*hht1*-*hhf1*)∆::*hhts-*  *Q120A*/*HHFS*-Hygro, (*hht2*-*hhf2*)∆::*hhts-Q120A/HHFS*-*URA3*,  *spt16-197* | This study |
| YS605 | *MAT***a** *ura3*∆*0*, *leu2*∆*0*, *trp1*∆*63*, (*hht1*-*hhf1*)∆::*hhts-Q120A*/*HHFS*- Hygro, (*hht2*-*hhf2*)∆::*hhts-Q120A*/*HHFS*-*URA3*, *spt16-197*,  *can1*∆::*MFA*pr-*HIS3* | This study |
| YS606 | *MAT***a** *ura3*∆*0*, *leu2*∆*0*, (*hht1*-*hhf1*)∆::*HHTS/HHFS*-Hygro, (*hht2*-  *hhf2*)∆::*HHTS/HHFS*-*URA3*, *spt2*∆*0*::KanMX | This study |
| YS607 | *MAT***a** *ura3*∆*0*, *leu2*∆*0*, (*hht1*-*hhf1*)∆::*HHTS/HHFS*-Hygro, (*hht2*-  *hhf2*)∆::*HHTS/HHFS*-*URA3*, *spt2*∆*0*::KanMX | This study |
| YS608 | *MAT*α *ura3*∆*0*, *leu2*∆*0*, (*hht1*-*hhf1*)∆::*HHTS/HHFS*-Hygro, (*hht2*-  *hhf2*)∆::*HHTS/HHFS*-*URA3*, *spt2*∆*0*::KanMX | This study |
| YS609 | *MAT*α *ura3*∆*0*, *leu2*∆*0*, *trp1*∆*63*, *his3*∆*200*, (*hht1*-*hhf1*)∆::*hhts- Q120A*/*HHFS*-Hygro, (*hht2*-*hhf2*)∆::*hhts-Q120A*/*HHFS*-*URA3*,  *spt2*∆*0*::KanMX | This study |
| YS610 | *MAT*α *ura3*∆*0*, *leu2*∆*0*, *trp1*∆*63*, *his3*∆*200*, (*hht1*-*hhf1*)∆::*hhts- Q120A*/*HHFS*-Hygro, (*hht2*-*hhf2*)∆::*hhts-Q120A*/*HHFS*-*URA3*,  *spt2*∆*0*::KanMX, *can1*∆::*MFA*pr-*HIS3* | This study |
| YS611 | *MAT***a** *ura3*∆*0*, *leu2*∆*0*, *trp1*∆*63*, *his3*∆*200*, (*hht1*-*hhf1*)∆::*hhts- Q120A*/*HHFS*-Hygro, (*hht2*-*hhf2*)∆::*hhts-Q120A*/*HHFS*-*URA3*,  *spt2*∆*0*::KanMX | This study |
| YS612 | *MAT***a** *ura3*∆*0*, *leu2*∆*0*, *trp1*∆*63*, *his3*∆*200*, *lys2*∆*0*, (*hht1*- *hhf1*)∆::*hhts-R49A*/*HHFS*-Hygro, (*hht2*-*hhf2*)∆::*hhts-R49A*/*HHFS*- *URA3*, *spt2*∆*0*::KanMX | This study |
| YS613 | *MAT*α *ura3*∆*0*, *leu2*∆*0*, *trp1*∆*63*, *his3*∆*200*, *lys2*∆*0*, *met15*∆*0*, (*hht1*- *hhf1*)∆::*hhts-R49A*/*HHFS*-Hygro, (*hht2*-*hhf2*)∆::*hhts-R49A*/*HHFS*- *URA3*, *spt2*∆*0*::KanMX | This study |
| YS614 | *MAT***a** *ura3*∆*0*, *leu2*∆*0*, *lys2*∆*0*, *his3*∆*200*, *met15*∆*0*, *trp1*∆*63* (*hht1*-  *hhf1*)∆::*hhts-R49A*/*HHFS*-Hygro, (*hht2*-*hhf2*)∆::*hhts-R49A*/*HHFS*-  *URA3*, FLAG-*spt6-1004* | This study |
| YS615 | *MAT***a** *ura3*∆*0*, *leu2*∆*0*, *lys2*∆*0*, *his3*∆*200*, *met15*∆*0*, (*hht1*-  *hhf1*)∆::*hhts-R49A*/*HHFS*-Hygro, (*hht2*-*hhf2*)∆::*hhts-R49A*/*HHFS*-  *URA3*, FLAG-*spt6-1004*, *RPB1*-C13Myc::KanMX | This study |
| YS616 | *MAT*α *ura3*∆*0*, *leu2*∆*0*, *lys2*∆*0*, *met15*∆*0*, (*hht1*-*hhf1*)∆::*hhts-R49A*/*HHFS*-Hygro, (*hht2*-*hhf2*)∆::*hhts-R49A*/*HHFS*-*URA3*, *spt16-*  *197* | This study |
| YS617 | *MAT***a** *ura3*∆*0*, *leu2*∆*0*, *lys2*∆*0*, (*hht1*-*hhf1*)∆::*hhts-R49A*/*HHFS*- Hygro, (*hht2*-*hhf2*)∆::*hhts-R49A*/*HHFS*-*URA3*, *spt16-197* | This study |
| YS618 | *MAT***a** *ura3*∆*0*, *leu2*∆*0*, *lys2*∆*0*, (*hht1*-*hhf1*)∆::*hhts-R49A*/*HHFS*-  Hygro, (*hht2*-*hhf2*)∆::*hhts-R49A*/*HHFS*-*URA3*, *spt16*-197 | This study |
| YS619 | *MAT***a** *ura3*∆*0*, *leu2*∆*0*, *trp1*∆*63*, *his3*∆*200*, (*hht1*-*hhf1*)∆::*hhts- R49A*/*HHFS*-Hygro, (*hht2*-*hhf2*)∆::*hhts-R49A/HHFS*-*URA3*, *spt2*∆*0*::KanMX | This study |
| YS640 | *MAT*α *his3*∆*200*, *lys2*∆*0*, *leu2*∆*0*, *ura3*∆*0*, (*hht1*-*hhf1*)∆::*hhts-K122A*- Hygro, (*hht2*-*hhf2*)∆::*hhts-K122A*-KanMX | This study |
| YS641 | *MAT***a** *his3*∆*200*, *lys2*∆*0*, *leu2*∆*0*, *ura3*∆*0*, (*hht1*-*hhf1*)∆::*hhts-K122A*- Hygro, (*hht2*-*hhf2*)∆::*hhts-K122A*-KanMX | This study |
| YS642 | *MAT*α *ura3*∆*0*, *leu2*∆*0*, *his3*∆*200*, *spt2*∆*0* | This study |
